# Pharmacological inhibition of tyrosine protein-kinase 2 reduces islet inflammation and delays type 1 diabetes onset in mice

**DOI:** 10.1016/j.ebiom.2025.105734

**Published:** 2025-05-06

**Authors:** Farooq Syed, Olivia Ballew, Chih-Chun Lee, Jyoti Rana, Preethi Krishnan, Angela Castela, Staci A. Weaver, Namratha Shivani Chalasani, Sofia F. Thomaidou, Stephane Demine, Garrick Chang, Alexandra Coomans de Brachène, Maria Ines Alvelos, Eugenia Martin Vazquez, Lorella Marselli, Kara Orr, Jamie L. Felton, Jing Liu, John S. Kaddis, Piero Marchetti, Arnaud Zaldumbide, Donalyn Scheuner, Decio L. Eizirik, Carmella Evans-Molina

**Affiliations:** a Indiana University School of Medicine, Indianapolis, IN, USA; b Center for Diabetes and Metabolic Diseases, Indiana University School of Medicine, Indianapolis, IN, USA; c Department of Pediatrics and the Herman B Wells Center for Pediatric Research, Indiana University School of Medicine, Indianapolis, IN, USA; d Indiana Biosciences Research Institute, Indianapolis, IN, USA; e ULB Center for Diabetes Research, Medical Faculty, Université Libre de Bruxelles, Brussels, Belgium; f Department of Cell and Chemical Biology, Leiden University Medical Center, the Netherlands; g Department of Physics, Indiana University Indianapolis, Indianapolis, IN, USA; h Department of Physics and Astronomy, Purdue University, West Lafayette, IN, USA; i Department of Medicine, Indiana University School of Medicine, Indianapolis, IN, USA; j Department of Biochemistry and Molecular Biology, Indiana University School of Medicine, Indianapolis, IN, USA; k Richard L. Roudebush VA Medical Center, Indianapolis, IN, USA; l Department of Translational Research and New Technologies in Medicine and Surgery, University of Pisa, Pisa, Italy; m Department of Clinical and Experimental Medicine, University of Pisa, Pisa, Italy; n Department of Diabetes-Immunology, Arthur Riggs Diabetes & Metabolism Research Institute, City of Hope, Duarte, CA, USA; o Department of Diabetes and Cancer Discovery Science, Arthur Riggs Diabetes & Metabolism Research Institute, City of Hope, Duarte, CA, USA

**Keywords:** Type 1 diabetes, β cell, T cell, Islets of langerhans, Tyrosine protein-kinase 2 (TYK2), Interferon-α

## Abstract

**Background:**

Tyrosine protein-kinase 2 (TYK2) mediates inflammatory signalling through multiple cytokines, including interferon-α (IFNα), interleukin (IL)-12, and IL-23. TYK2 missense mutations protect against type 1 diabetes (T1D), and inhibition of TYK2 shows promise in other autoimmune conditions.

**Methods:**

We evaluated the effects of specific TYK2 inhibitors (TYK2is) in pre-clinical models of T1D, including human β cells, cadaveric islets, iPSC-derived islets, and mouse models.

**Findings:**

*In vitro* studies showed that TYK2is prevented IFNα-induced β cell HLA class I up-regulation, endoplasmic reticulum stress, and chemokine production. In co-culture studies, pre-treatment of β cells with TYK2i prevented IFNα-induced antigenic peptide presentation and alloreactive and autoreactive T cell degranulation. *In vivo* administration of BMS-986202 in two mouse models of T1D (*RIP-LCMV-GP* and NOD mice) reduced systemic and tissue-localised inflammation, prevented β cell death, and delayed T1D onset. Transcriptional phenotyping of pancreatic islets, pancreatic lymph nodes, and spleen highlighted a role for TYK2 inhibition in modulating signalling pathways associated with inflammation, translational control, stress signalling, secretory function, immunity, and diabetes. Additionally, TYK2i treatment changed the composition of innate and adaptive immune cell populations in the blood and disease target tissues.

**Interpretation:**

These findings indicate that TYK2i has beneficial effects on both the immune and endocrine compartments in models of T1D, thus supporting a path forward for testing TYK2is in human T1D.

**Funding:**

This work was supported by the 10.13039/100000002National Institutes of Health (NIH), Veteran Affairs (VA), Breakthrough T1D, and gifts from the Sigma Beta Sorority, the 10.13039/100000989Ball Brothers Foundation, and the 10.13039/100014159George and Frances Ball Foundation.

**Research in context**

**Evidence before this study**

Type 1 diabetes (T1D) results from autoimmune destruction of insulin producing pancreatic β cells. Polymorphisms in tyrosine protein-kinase 2 (TYK2) are associated with a reduced risk of several autoimmune diseases, including T1D, ulcerative colitis, and rheumatoid arthritis. Additionally, pharmacological inhibition of another Janus kinase family member via baricitinib treatment was recently shown to preserve C-peptide in individuals who were newly diagnosed with Stage 3 T1D.

**Added value of this study**

We have used three *in vitro* human model systems and two *in vivo* preclinical mouse models to determine how pharmacological inhibition of TYK2 mediates crosstalk between the immune system and β cells in T1D. We found that TYK2 inhibitors (TYK2is) block IFN signalling *in vitro* and delay the onset of T1D *in vivo*. Detailed mechanistic studies determined that TYK2is impair activation of the innate and adaptive immune responses, reduce β cell inflammation and apoptosis, and prevent pathological interactions between T cells and β cells.

**Implications of all the available evidence**

Our studies add to the existing literature suggesting that inhibition of IFN-mediated signalling has beneficial effects on immune regulation and β cell function. Therefore, these findings support a path forward to test whether TYK2is could be a viable treatment to delay or prevent the onset of T1D in humans.

## Introduction

During the development of type 1 diabetes (T1D), cells of the innate and adaptive immune system infiltrate the pancreatic islets, produce a variety of pro-inflammatory cytokines, and create a feed-forward cycle of immune activation, inflammation, and β cell death. Tyrosine protein-kinase 2 (TYK2), a member of the Janus kinase (JAK) family, transduces signals from type I and type II cytokine receptors via phosphorylation and activation of the transcription factors, signal transducer and activator of transcription 1 and 2 (STAT1 and 2).^1^^,^^2^ Polymorphisms in TYK2 are associated with a reduced risk of several autoimmune diseases, including T1D, ulcerative colitis, and rheumatoid arthritis.^3^, ^4^, ^5^ Notably, a polymorphism causing a missense mutation in TYK2, leading to decreased function, is associated with protection against T1D.^4^^,^^6^^,^^7^

TYK2 modulates signalling through three specific cytokines: interferon-α (IFNα), interleukin (IL)-12, and IL-23.^8^^,^^9^ While each of these cytokines has been linked with T1D pathogenesis, clinical and preclinical data suggest a prominent role for IFNα in mediating inflammatory crosstalk between β cells and the immune system during T1D progression. There is evidence of a type 1 interferon transcriptional signature in the peripheral blood of children at high genetic risk for T1D even prior to seroconversion^10^^,^^11^ and in the whole blood and pancreas of individuals with recent onset T1D.^12^ IFNα activates pancreatic islet-resident macrophages and T cells, while also impacting stress pathways in the β cell.^13^, ^14^, ^15^, ^16^ Findings by our group and others have shown that treatment of human β cells with IFNα augments HLA class I overexpression, modulates transcriptional programs associated with endoplasmic reticulum (ER) stress and inflammatory signalling, and increases alternative RNA splicing and the diversity of transcripts expressed by β cells.^17^, ^18^, ^19^, ^20^, ^21^ Furthermore, case reports have documented T1D onset in conjunction with the therapeutic use of IFNα in hepatitis C and leukaemia.^22^^,^^23^ In contrast, antibody-mediated blockade of IFNα or the type I IFN α/β receptor (IFNAR1) is sufficient to prevent the development of T1D in rodent models.^24^, ^25^, ^26^, ^27^

Blocking IFNα-mediated effects via inhibition of JAK tyrosine kinases has shown beneficial effects in *in vivo* murine and *in vitro* cell models of T1D.^28^^,^^29^ Additionally, in a recent clinical trial, the JAK1/2 inhibitor baricitinib preserved C-peptide in individuals 10–30 years of age who were newly diagnosed with Stage 3 T1D.^30^ Pharmacological inhibition of TYK2 may offer safety advantages over JAK inhibitors, which are associated with an increased risk of venous and arterial thrombotic events, cardiovascular events, and malignancy in older populations.^31^^,^^32^ In 2022, a selective allosteric inhibitor targeting the TYK2 pseudokinase domain (deucravacitinib; BMS-986165) was approved by the U.S. Food and Drug Administration (FDA) for use in psoriasis.^33^ Previous *in vitro* studies suggest TYK2 inhibitors are able to block IFNα-mediated signalling in human islets and β cell lines.^34^^,^^35^ In addition, a recent study showed that NOD mice with total body TYK2 deletion were protected from the development of T1D.^36^

To define how pharmacological TYK2 inhibition mediates *in vivo* crosstalk between the immune system and the β cells, we tested the effects of two TYK2is, BMS-986165 and BMS-986202, in three *in vitro* human model systems and then determined the impact of BMS-986202 in two mouse models of T1D.^37^^,^^38^ We demonstrate that allosteric TYK2 inhibition blocked IFN-mediated transcriptional responses *in vitro* and delayed the onset of diabetes in preclinical murine models of T1D. Mechanistic studies, including *in situ* spatial transcriptomics, defined a phenotype whereby pharmacological inhibition of TYK2: 1) dampens innate immune cell activation, 2) impairs adaptive immune responses, 3) directly reduces β cell inflammation and apoptosis, and 4) prevents pathological interactions between T cells and β cells. Taken together, these data support a path forward for testing TYK2is in human T1D.

## Methods

### Animal studies

*RIP-LCMV-GP* mice were purchased from Jackson Laboratory and bred in-house. Eight-week-old male *RIP-LCMV-GP* mice were pre-treated with TYK2i (BMS-986202, 30 mg/kg) or vehicle (100 μL of 0.5% Methyl Cellulose-4M per mouse) by oral gavage two days prior to inoculation with LCMV. Mice continued to receive TYK2i treatment daily until the study endpoint. To monitor for T1D development, blood glucose levels were measured before inoculation and on days 1, 4, 7, 11, and 14 post-inoculation, and two consecutive blood glucose readings over 250 mg/dL established diabetes development.

Five-week-old female NOD/ShiLT mice were purchased from Jackson Laboratory and acclimated at the IU Laboratory Animal Resource Center (LARC) for at least one week prior to treatment. Six-week-old female NOD mice were treated with BMS-986202 (30 mg/kg) or vehicle for 6 weeks by oral gavage and followed for up to 24 weeks to determine diabetes development. Blood glucose levels were measured weekly between ages 6–14 weeks and biweekly from 15 to 24 weeks of age. Two consecutive blood glucose readings over 250 mg/dL established diabetes development. To investigate insulitis, a subset of mice from each group was euthanised after 6 weeks of TYK2i treatment (12-weeks of age), and the pancreas was harvested and fixed for downstream analysis as described previously.^39^

### Sex as a biological variable

In our study, we considered sex to be a biological variable. Due to the cumulative diabetes incidence in NOD female mice being 80%, compared to less than 20% in male mice,^40^ we exclusively used female NOD mice for our investigation. Additionally, we noted a 100% penetrance of diabetes only in male RIP-LCMV-GP mice compared to female mice. Therefore, we focused solely on male RIP-LCMV-GP mice for this study.

### Culture and treatment of human EndoC-βH1 cells and human islets

Human insulin-secreting EndoC-βH1 cells^41^ were cultured in Matrigel-fibronectin-coated plates as previously described.^20^ Cells are monitored continuously for doubling times and morphology. They are tested every 9–12 months to ensure identity and to verify that they are free of contamination from other cell lines and microbes. The presence of mycoplasma infection is surveilled using the MycoAlert Mycoplasma Detection kit (Lonza, catalogue #LT07-318). Cells are periodically refreshed from frozen stocks to maintain a low passage number. Human islets from 8 organ donors without diabetes were isolated by collagenase digestion and density gradient purification for dispersion and experiments.^20^

All experiments with EndoC-βH1 cells or human islets are shown as independent biological data (i.e.*,* considering EndoC-βH1 cells from different passages or human islets from different donors as n = 1). Cells were treated with the TYK2i BMS-986165 (MedChemExpress, catalogue #HY-117287) or BMS-986202 (a kind gift from Bristol Myers Squibb) at different concentrations ranging from 0.0003 to 3 μM and with different proinflammatory cytokines alone or in combination: IFNα (2000 U/mL, PeproTech, catalogue #300-02AA-1MG), IL-1β (50 U/mL, R&D Systems, catalogue #201-LB-005), and TNFα (1000 U/mL, PeproTech, catalogue #300-01A-50UG). Cytokine dosing and experimental endpoints were based on previous time-course analysis performed by our group.^21^^,^^42^, ^43^, ^44^ Brefeldin A (0.02 μg/mL, Sigma Aldrich, catalogue# B6542) and thapsigargin (1 μM, Sigma Aldrich, catalogue #T9033) were used to determine the effect of TYK2 inhibition on chemically induced ER stress.

#### Differentiation of iPSCs into β cells

Cells were differentiated into pancreatic β-like cells using a 7-step protocol.^45^ After differentiation, stage 7 aggregates were dispersed^20^ for further treatment with TYK2i and/or cytokines. iPSC lines must follow a pattern consistent with pancreatic, endocrine, and β cell development, reaching mRNA levels of insulin and Pdx1 that are overall comparable to primary human islets, usually resulting in >50% β cells and few α- or insulin- and glucagon-double positive cells. A normal karyotype (i.e. 46 XY or XX) is confirmed before use. Islet identity and quality are assessed in a stepwise fashion by performing PDX1 and NKX6.1 staining to confirm pancreatic progenitor identity, performing imaging to assess the distinctness of insulin and glucagon staining in mature islet clusters, and by verifying glucose stimulated insulin secretion. Cultured lines are limited to 20 total passages and are continuously monitored for doubling times and morphology.

#### Assessment of cell viability

Cells were stained with the DNA-binding dyes propidium iodide (PI) (Sigma–Aldrich, catalogue #P4170) and Hoechst 33342 (10 μg/mL, Sigma–Aldrich, catalogue #94403-1 ML) to count apoptotic cells by fluorescent microscopy, as previously described.^46^ This method is quantitative and has been validated by systematic comparison against electron microscopy^47^ and other well-characterised methods to determine apoptosis, such as fluorometric caspase 3 & 7 assays and determination of histone-complexed DNA fragments by ELISA.^48^

### mRNA extraction and quantitative real-time PCR

Cells were washed with PBS, and polyadenylated mRNA was extracted using the Dynabeads mRNA DIRECT kit (Invitrogen, catalogue #61011) and reverse transcribed with the Reverse Transcriptase Core kit (Eurogentec, catalogue #RT-RTCK-03). Gene expression was analysed by quantitative real-time PCR (qRT-PCR) using the SsoAdvanced Universal SYBR Green Supermix (BIO-RAD, catalogue #1725270). Expression of the genes of interest was detected with the following primers (5′-3′): *HLA-ABC* fwd: CAGGAGACACGGAATGTGAA; *HLA-ABC* rev: TTATCTGGATGGTGTGAGAACC; *DDIT3(CHOP)* fwd and rev: Qiagen QuantiTect primer; *ATF3* fwd: GCTGTCACCACGTGCAGTAT; *ATF3* rev: TTTGTGTTAACGCTGGGAGA; *CXCL10* fwd: GTGGCATTCAAGGAGTACCTC; *CXCL10* rev: GCCTTCGATTCTGGATTCAG; *MX1* fwd: AGACAGGACCATCGGAATCT; *MX1* rev: GTAACCCTTCTTCAGGTGGAAC. *INS* fwd: CCAGCCGCAGCCTTTGTGA; *INS* rev: CCAGCTCCACCTGCCCCA; *PDX1* fwd: AAAGCTCACGCGTGGAAA; *PDX1* rev: GCCGTGAGATGTACTTGTTGA; *PD-L1* fwd: CCAGTCACCTCTGAACATGAA; *PD-L1* rev: ACTTGATGGTCACTGCTTGT; *B2M* fwd: TCGCGCTACTCTCTCTTTCTG; *B2M* rev: TTCTCTGCTGGATGACGTGAG; ATF3 fwd: GCTGTCACGTGCAGTAT; ATF3 rev: TTTGTGTTAAGCTGGGAGA; BIP (Qiagen, quantiTect primer Hs_HSPA5_1_SG, catalogue #QT00096404). The quantification of amplicons was done with a standard curve, and gene expression was normalised by the expression of the reference gene *ACTB* (fwd: CTGTACGCCAACACAGTGCT; rev: GCTCAGGAGGAGCAATGATC).

### Protein extraction and Western blot analysis

Cells were lysed with 1x Laemmli Sample buffer (60 mM tris(hydroxymethyl)aminomethane, pH 6.8, 10% Glycerol, 2% Sodium dodecyl sulphate, 1.5% 2-mercaptoethanol, 1.5% Dithiothreitol, and 0.005% bromophenol blue) containing phosphatase and protease inhibitors (PhosSTOP and cOmplete ULTRA tablets, mini, EASYpack protease inhibitor cocktail, Roche, catalogue #04693132001), and proteins were analysed by Western blot. To evaluate STAT1 and STAT2 activation, we measured their phosphorylation using a rabbit anti-phospho-STAT1 (1:1000, Cell Signalling Technology, catalogue #9167, RRID:AB_561284) and a rabbit anti-phospho-STAT2 antibody (1:4200, Cell Signalling Technology Cat# 88410, RRID:AB_2800123). In experiments that used chemical ER stress inducers, total STAT1 (Cell Signalling, cat# 9176S) was used to assess the activation status of pSTAT1. Immunoreactive bands were detected using ChemiDoc XRS+ (Bio-Rad) after incubation with a secondary anti-rabbit antibody coupled with horseradish peroxidase (HRP, 1:5000, Thermo Fisher Scientific Cat# 32460, RRID:AB_1185567) and exposure to the SuperSignal West Femto chemiluminescent substrate (Thermo Fisher Scientific, catalogue #34094). Densitometric quantification of the bands was performed with ImageLab software (Bio-Rad). Data were normalised for the expression of the housekeeping protein β-actin (1:5000, (Cell Signalling Technology Cat# 4967, RRID:AB_330288).

#### Generation of EndoC-βH1 cells expressing HLA-A2 and T cell activation assays

EndoC-βH1 cells expressing HLA-A2 were generated by lentivirus transduction^49^ using a lentiviral vector containing HLA-A02:01 under the elongation factor 1α (EF1α) promotor.^50^ Target cells (i.e. EndoC-βH1 cells expressing HLA-A2) were harvested and cocultured with HLA-A2 alloreactive cytotoxic lymphocytes (CTLs)^51^ or autoreactive T cells directed against preproinsulin^52^ and insulin-DRiP.^53^ Cocultures between CTLs and EndoC-βH1 cells expressing HLA-A2 were performed at 37 °C for 1.5 h in Iscove's Modified Dulbecco's Medium (IMDM) supplemented with 10% human serum and 25 U/mL IL-2 (Novartis), as described previously.^54^^,^^55^ The supernatant was used for detection of macrophage inflammatory protein-1β (MIP-1β) production by T cells using the MIP-1β ELISA kit (Thermo Fisher Scientific, catalogue #88-7034-22, RRID:AB_2574957), according to the manufacturer's protocol.

### Flow cytometry

Spleen, pancreatic lymph nodes (PLNs), and blood were collected from TYK2i- or vehicle-treated *RIP-LCMV-GP* mice. Spleen and PLNs were homogenised and passed through a 70 μm cell strainer (BD Falcon, San Jose, CA) to obtain a single-cell suspension. Cells were incubated with mouse anti-CD16/32 (BD Biosciences catalogue #553142, RRID:AB_394657) to block Fc receptors and then surface-stained with fluorochrome-conjugated monoclonal antibodies for 20 min at 4 °C followed by PBS wash. Live/dead staining was performed using Zombie aqua (1:1000 dilution, BioLegend) for 20 min at room temperature to exclude dead cells. Multivariable flow cytometry was performed using anti-mouse monoclonal antibodies: CD3-FITC (17A2) (Thermo Fisher Scientific, catalogue #14-0032-82, RRID:AB_467053), CD19-A700 (1D3) (BioLegend, catalogue #152413, RRID:AB_2922474), CD11b-BV650 (M1/70) (BioLegend, catalogue #101259, RRID:AB_2566568), CD11c-APC (N418) (BioLegend, catalogue #117310, RRID:AB_313779), MHCII-PerCPCy5.5 (M5/114.15.2) (BioLegend, catalogue #107626, RRID:AB_2191071), F4/80-PE (BM8) (BioLegend, catalogue #123110, RRID:AB_893486), Ly6c-PECy7 (HK1.4) (BioLegend, catalogue #128018, RRID:AB_1732082), CD49b-APCCy7 (DX5) (BioLegend, catalogue #108920, RRID:AB_2561458), CD4-A700 (GK1.5) (BioLegend, catalogue #100430, RRID:AB_493699), CD8a-PerCPCy5.5. (53-6.7) (BioLegend, catalogue #100734, RRID:AB_2075238), CD25-BV650 (PC61) (BioLegend, catalogue #102038, RRID:AB_2563060), CD44-APCCy7 (IM7) (BioLegend, catalogue #103028, RRID:AB_830785), CD62L-PE (MEL-14) (BioLegend, catalogue #104408, RRID:AB_313095), CD69-PECy7 (H1.2F3) (BioLegend, catalogue #104512, RRID:AB_493564), PD1-BV605 (29F.1A12) (BioLegend, catalogue #135220, RRID:AB_2562616), and CXCR3-BV421 (S18001A) (BioLegend, catalogue #155907, RRID:AB_2832543). Single cell suspensions were permeabilised for intracellular staining using FoxP3 fix/perm buffer (Thermo Fisher Scientific, catalogue #00-5523-00) at 4 °C for 20 min, followed by a wash with permeabilisation buffer. Samples were then stained with mouse anti-FoxP3-APC (FJK-16s; Thermo Fisher Scientific, catalogue #50-5773-82, RRID:AB_11218868) for 30 min at 4 °C, followed by PBS wash. Cell suspensions were evaluated on an Attune NxT flow cytometer (Thermo Fisher Scientific), and all analysis was performed in FCS Express (Version 7, De Novo Software).

### Immunohistochemistry

Pancreatic tissues were harvested and fixed with 4% PFA overnight at 4 °C and tissues were processed and embedded in paraffin blocks as described previously.^39^ Tissue sections were stained with rabbit anti-insulin antibody (Cell Signalling Technology, catalogue #3014S, RRID:AB_2126503) and counterstained with DAB peroxidase anti-rabbit IgG (Vector Laboratories, catalogue #PI-1000-1, RRID:AB_2916034) Images were acquired using a Zeiss slide scanner (Zeiss, Germany), and insulitis scoring was performed on 5 slides, each 30 μm apart, from 9 mice/group as previously described.^39^ Immunofluorescence studies were performed according to the protocol described previously^56^ using the following antibodies: guinea pig anti-insulin (Agilent, catalogue #IR002, RRID:AB_2800361), mouse anti-PD-L1 (Proteintech, catalogue #66248-1-Ig, RRID:AB_2756526), and rabbit anti-CXCL10 (Thermo Fisher Scientific, catalogue #701225, RRID:AB_2532429). Signals were detected by counter staining with the following antibodies: goat anti-guinea pig (1:400; Molecular Probes, catalogue # A-11073, RRID:AB_2534117), goat anti-rabbit (1:200; Molecular Probes, catalogue #A-21244, RRID:AB_2535812), and goat anti-mouse (1:200; Molecular Probes, catalogue #A-11031, RRID:AB_144696). All sections were counterstained with DAPI to identify nuclei. Images were obtained with a Zeiss LSM 800 confocal microscopy (Carl Zeiss, Germany), the fluorescence intensities were quantified using Image J, and the corrected fluorescence intensities were presented, as described previously.^56^ A detailed list of antibodies used in this study, along with their associated RRIDs, is provided in Table 1.

**Table 1:** Reagent list.

Reagent or resource	Source	Identifier
**Animals and cell lines**		
NOD/ShiLtJ	Jackson Laboratory	Cat#001976; RRID:IMSR_JAX:001976
B6.C-Tg (Ins2-GP)34-20Olds/Mvh	Jackson Laboratory	Cat#005500; RRID:IMSR_JAX:005500
EndoC-βH1-HLA-A2 cells	University Medical Center Utrecht	
EndoC-βH1	Human Cell Design	RRID:CVCL_L909
**Chemicals and materials**		
Iscove's Modified Dulbecco's Medium (IMDM)	ThermoFisher Scientific	Cat#12440053
Dulbecco's Modified Eagle Medium	ThermoFisher Scientific	Cat#11885-084
Foxp3/Transcription Factor Staining Buffer Set	eBioscience™	Cat#00-5523-00
SuperSignal™ West Pico PLUS Chemiluminescent Substrate	ThermoFisher Scientific	Cat#34577
ProLong™ Glass Antifade Mountant	ThermoFisher Scientific	Cat#P36984
Methyl Cellulose-4M	Sigma	Cat#434965
BMS-986165	MedChemExpress	Cat#HY-117287
MycoAlert® Mycoplasma Detection Kit	Lonza	Cat #: LT07-318
PhosSTOP and cOmplete ULTRA tablets	Roche	Cat#04906837001
EASYpack protease inhibitor cocktail	Roche	Cat#04693132001
Recombinant human IL-2	Proleukin, Novartis, Switzerland	
Recombinant human IFNα	PeproTech	Cat# 300-02AA-1MG
Recombinant human IL-1β	R&D Systems	Cat#201-LB-005
Recombinant human TNFα	PeproTech	Cat#300-01A-50UG
SsoAdvanced Universal SYBR Green Supermix	Bio-Rad	Cat#1725270
Propidium iodide	Sigma	Cat#P4170
RNAscope Probe- Hs-CD274	Advanced Cell Diagnostics	Cat#420501-C2
RNAscope Probe- Hs-CXCL10	Advanced Cell Diagnostics	Cat#408921-C3
RNAscope Probe- Hs-Mx1	Advanced Cell Diagnostics	Cat#474931
RNAscope Probe- Hs-STAT1	Advanced Cell Diagnostics	Cat#479611-C2
RNAscope Multiplex Fluorescent Detection kit	Advanced Cell Diagnostics	Cat#323110
HC Antigen Retrieval Solution–High pH (10X)	ThermoFisher Scientific	cat#SP-5030; RRID:AB_2336107
GMX-RNA-NGS-MsWTA	NanoString Technologies	Cat#121401103
GeoMx Seq Code Pack_AB	NanoString Technologies	Cat#121400201
GeoMx Nuclear Stain Morp Kit	NanoString Technologies	Cat#121300303
GeoMx Instrument Buff Kit PCLN	NanoString Technologies	Cat#100474
Syto83	Invitrogen	cat#S11364
Normal Donkey Serum	Jackson ImmunoResearch laboratories Inc	Cat#017-000-121; RRID: AB_2337258
Dynabeads mRNA DIRECT kit	Invitrogen	Cat#61011
Hoechst 33342	Sigma–Aldrich	Cat#94403-1 ML
Reverse Transcriptase Core Kit	Eurogentec	Cat#RT-RTCK-03
Brefeldin A	Sigma Aldrich	Cat#B6542
Thapsigargin	Sigma Aldrich	Cat#T9033
**Software and algorithms**		
GraphPad Prism version 10.1.0	GraphPad	RRID: SCR_002798
FIJI- Image J	https://imagej.nih.gov/ij/	RRID: SCR_003070
FCS Express (Version 7)	De Novo software	RRID:SCR_016431
GeomxTools 3.4.0 package	NanoString Technologies	RRID:SCR_023912
Ingenuity Pathway Analysis	Qiagen	RRID:SCR_008653
GeoMx DSP analysis suite (Version 3.0.109)	NanoString Technologies	RRID:SCR_023912
Image Lab software	Bio-Rad	Cat#17006172
SpatialDecon package	(http://www.bioconductor.org/packages/release/bioc/manuals/SpatialDecon/man/SpatialDecon.pdf)	

#### Single-molecule fluorescence *in situ* hybridisation

PFA fixed and paraffin-embedded tissue sections cut under RNase-free conditions were pre-treated and processed as described in our previous studies.^57^^,^^58^ Fluorescence *in situ* hybridisation was performed using an RNAscope Multiplex Fluorescent Detection kit (RNAscope) according to the manufacturer's protocol. Briefly, a combination of two probes targeting mouse genes (*CD274* and *Mx1* or *Cxcl10* and *Stat1* at day 3 and day 14*;* and *CD274* and *Cxcl10* or *Mx1* and *Stat1* at day 7) was hybridised for 2 h, and the signals were detected using secondary TSA fluoroprobes using the cyclic detection method for two different channels with co-staining for insulin and DAPI. The hybridised sections were mounted using ProLong Antifade mounting media (Thermo Fisher Scientific, catalogue #P36984) and imaged using a Zeiss LSM800 microscope (Carl Zeiss, Germany) attached to an Airyscan detector.

#### smFISH analysis

The processing, analysis, and quantification of smFISH images was performed similar to our previous work—*see preprint*,^59^ which involves a series of image detection algorithms and data processing tools. Initially, the multi-channel images were segregated into four distinct channels, including the nucleus channel, the insulin protein channel, and two mRNA channels. We used a combination of manual segmentation and the implementation of the Segment Anything Model (SAM)—*see preprint*^60^ to identify the nucleus from the DAPI channel. Post-processing of the SAM-segmented nucleus masks involved the removal of duplicate masks and the elimination of segmented objects with areas smaller than 3000 pixels (approximately 2.7 μm^2^). Subsequently, the nuclear boundary was extended radially to predict the cell boundary. The identification of β cells was achieved by overlaying the segmented cells with the insulin protein channel, allowing for the recording of insulin protein intensity for each cell. To further analyse the β and non-β cell populations, a histogram was plotted based on the resulting insulin protein intensity per cell. Savitzky–Golay filter was applied to smooth the histogram by yielding two distinct cell populations. By identifying the local minima in the smoothed histogram, the separation between distributions was determined, establishing the insulin intensity threshold for β cell identification.

To detect the individual mRNA particles from the two mRNA channels, the Laplacian of Gaussian (LoG) algorithm was utilised.^61^ This algorithm provides estimated locations of mRNA particles, facilitating subcellular expression analysis. In some instances, multiple mRNA particles may exhibit intense brightness due to overlapping at close proximity, showing a single bright focus on smFISH images. To address this issue, the number of mRNAs at a bright focus was deduced by normalising the focus intensity of the single mRNA intensity.^62^ The intensity of a single mRNA was set by binning mRNA particles based on intensity and identifying the intensity bin containing the majority of mRNAs. Finally, outliers caused by autofluorescence were further filtered out using Tukey's fence^63^ to prevent overcounting of RNAs.

#### Nanostring GeoMx DSP spatial transcriptomics

Mouse pancreas sections (5 μm) were fixed, sectioned, and mounted on positively charged slides. The slides were deparaffinised, heated in antigen retrieval solution (Thermo Fisher Scientific, catalogue #00-4956-58) at 100 °C for 20 min, and treated with 1 μg/mL proteinase K at 37 °C for 15 min. Mouse transcriptomics was visualised using the GeoMx mouse whole transcriptome atlas (NanoString Technologies, GMX-RNA-NGS-MsWTA). An overnight *in situ* hybridisation was performed with a mouse WTA probe, and the next day, the slides were washed twice at 37 °C for 25 min with 50% formamide/2X SSC buffer to remove unbound probes. The slides were then washed twice with 2X SSC buffer for 2 min at RT and blocked with blocking buffer containing Buffer W (Nanostring) with 1% Fc-Receptor blocker (Milteny) and 5% Normal Donkey Serum (Jackson Laboratories) as described previously.^64^ The slides were incubated with guinea pig anti-insulin (Agilent, catalogue #IR002, RRID:AB_2800361), 1:200 rabbit anti-CD3 (Abcam, catalogue #ab135372, RRID:AB_2884903), mouse anti-CD68 (Santa Cruz Biotechnology, catalogue #sc-20060, RRID:AB_627158). The slides were washed twice with 2X SSC and incubated with secondary antibodies (1:400; goat anti-guinea pig Alexa-488, Molecular Probes, catalogue #A-11073, RRID:AB_2534117); 1:200 goat anti-rabbit Alexa-594 (Molecular Probes, catalogue #A-11072, RRID:AB_142057), and 1:200 goat anti-mouse Alexa-647 (Thermo Fisher Scientific, catalogue #A-21238, RRID:AB_2535807) for 30 min. Then, the slides were washed four times with 2X SSC for 3 min and counter-stained with 1:20,000 Syto83 (Invitrogen, catalogue #S11364) for 1 h at room temperature. Stained slides were loaded onto the GeoMx instrument and scanned. After visual inspection using the GeoMx Digital Spatial Profiler (DSP; NanoString Technologies), geometrical region of interest (ROIs) (100 μm diameter) with different histologies were selected for oligonucleotide collection. Photocleaved oligonucleotides from each spatially resolved ROI were sequenced with the Illumina NextSeq500 sequencer (Illumina). The GeoMx libraries were prepared according to the GeoMx DSP NGS Readout User Manual. Briefly, for each plate, 4 μL of a GeoMx DSP sample was used for amplification. Each GeoMx DSP sample in a well was uniquely indexed with dual indexes. Four μL of each resulting PCR product, including the NTC negative control, were pooled and purified with AMPure XP beads (Beckman Coulter, catalogue #A63881). The final libraries were qualified and quantified using TapeStation and Qubit, respectively. The libraries were sequenced on an Illumina NextSeq 500 to generate 27 bp paired-end sequence reads.

#### Spatial transcriptomics data analysis

Spatial transcriptomics analysis was conducted in R studio v4.2.1 using the GeomxTools 3.4.0 package.^65^ The GeoMx whole mouse transcriptome atlas was used as the probe assay metadata. Sequencing quality was assessed using the default quality control (QC) parameter cut-offs, namely: minimum number of reads (1000); minimum percentage of reads trimmed (80%), stitched (80%), and aligned (80%); sequencing saturation (50%); negative control counts(10); maximum counts observed in no treatment control (NTC) well (1000); minimum number of nuclei estimated(100); and minimum segment area (5000). Any ROI that did not satisfy the above criteria was removed as part of QC filtering. Following segment QC, probe QC was also performed to remove any outlier either globally or locally before generating gene-level count data. The limit of quantification was determined per segment using the value recommended in the workflow, and segments with less than 1–5% of genes detected were removed. Furthermore, only genes that were detected in at least 1% of segments were retained. The data was normalised using the quartile 3 method and log transformed. Samples were clustered based on overall gene expression using either UMAP (umap package) or tSNE (Rtsne package). Genes with a high coefficient of variation were plotted using unsupervised hierarchical clustering displayed as a heatmap. For each organ, a linear mixed model without random slope was used to identify differentially expressed genes between vehicle and treatment groups for both the 3- and 7-day timepoints. Genes with linear scale fold change ≥1.5 and P-value <0.05 were considered as differentially expressed. These differentially expressed genes were visualised using volcano plots, and the top ten differentially expressed genes from each region were visualised using heatmaps (pheatmap function from gplots package). All graphics were generated using ggplot2 unless otherwise stated. Pathway analysis for each time point from each organ was performed using the Qiagen Ingenuity pathway analysis tool (QIAGEN Inc., https://digitalinsights.qiagen.com/IPA). Pathways with a z-score of at least 1 and P-value <0.05 were considered for further interpretation. Spatial deconvolution for each organ and every time point was performed using the SpatialDecon package (http://www.bioconductor.org/packages/release/bioc/manuals/SpatialDecon/man/SpatialDecon.pdf). Normalised values obtained from the above workflow were used as the input for the expression matrix. Adult mouse pancreas (Pancreas_MCA) and adult mouse spleen (Spleen_MCA) were used as cell matrices for determining the cell populations in the pancreas and spleen, respectively. In addition, the mouse immune profile (ImmuneAtlas_ImmGene) was used as the cell matrix for estimating immune cell abundance in the pancreas, spleen, and PLN. The cell matrices were downloaded from.^66^^,^^67^ Basic deconvolution was performed following the workflow provided in the R package. For immune cell populations, the cells were merged based on the lineage. Beta values were used as the input for heatmap (pheatmap function from gplots package) and abundance plots. Any population with a zero value was removed from the graphs. A Shapiro Wilks test was used to assess normality and an F-test for homogeneity of variance between groups if the data was normally distributing. If not, non-parametric statistical tests were used to evaluate differences. Stacked bar charts illustrating the abundance of cell populations and statistical analysis was performed using GraphPad Prism.

#### Spatial proteomics using the NanoString GeoMx spatial profiling platform

Pancreatic sections were deparaffinised and rehydrated, similar to the transcriptomic steps described above. Next, antigen retrieval was performed with 1X citrate buffer pH 6.0 in a preheated pressure cooker for 15 min. The slides were then stained with anti-insulin (AlexaFluor 488, 1:50; R&D Systems, catalogue #IC1417G, RRID:AB_3654708), anti-B220 (AlexaFluor 594, 1:150; BioLegend, catalogue #103254, RRID:AB_2563229)), and anti-CD3 (AlexaFluor 647, 1:200; Bio-Rad, catalogue #MCA1477A647, RRID:AB_10841760) antibodies. In addition, DSP antibody barcode collections including immune cell core profiling, immune cell activation, and immune cell typing (nanoString) were added to the slides and incubated overnight at 4 °C. Nuclei were identified by staining with Syto83 fluorescent dye (0.2 μm concentration, Fluorescence 532, Thermo Fisher Scientific). The corresponding ROIs from the protein slide were selected and collected in a collection plate using a similar method as the RNA profiling. The aspirates were then reacted directly with Probe R and Probe U (Nanostring Technologies), which carry the fluorescent reporters and can be used for fluorescence counting with the nCounter Max Analysis system (Nanostring Technologies, Inc).

#### GeoMx DSP proteomic data analysis

Protein expression counts from the nCounter run were retrieved and uploaded into the GeoMx DSP analysis suite (Nanostring, version 3.0.109), and the nCounter read counts were normalised using a geometric mean of the signal-to-noise ratio (SNR) of IgG negative controls as described previously.^64^^,^^68^ The normalised read counts are presented as Log_2_ of SNR, and differences between groups are presented with associated P values.

### Ethics

Mouse studies were conducted in accordance with Animal Research: Reporting of *In Vivo* Experiments (ARRIVE) guidelines under protocols approved by the Indiana University (IU) School of Medicine Animal Use Committee. NOD and *RIP-LCMV-GP* mice were maintained at the IU School of Medicine Laboratory Animal Resource Center (LARC), and the protocol was approved by the IU School of Medicine Institutional Animal Care and Use Committee (IACUC, current protocol number: 23154 MD/R/HZ/E; previous protocol number: 20104 MD/R/HZ/E). The iPSC lines HEL46.11 and HEL115.6 were derived from fibroblasts from neonatal foreskin^69^ or umbilical cord^45^ obtained from healthy human donors after informed consent and approval by the Ethics Committees of the Helsinki and Uusimaa Hospital District (Helsinki, Finland) and the Erasmus Hospital (ULB, Brussels, Belgium). Human islets from 8 organ donors without diabetes were isolated in Pisa, Italy by collagenase digestion and density gradient purification with the approval of the local ethical committee and sent to Universite Libre de Bruxelles (ULB) for dispersion and experiments.^20^

#### Statistics

*Sample size determination:* For experiments using NOD mice, we calculated the sample size using several considerations: 1) 75–80% of female mice develop diabetes if left untreated, and 2) preliminary data on TYK2 inhibitors from published studies^36^ indicate a ∼80% reduction in diabetes incidence in NOD mice treated with TYK2 inhibitors. Therefore, at a power of 0.80, an alpha 0.05, and using a 1-sided fishers exact conditional test, a minimum of 10 mice are needed in each group (vehicle and TYK2-treated mice) to detect this difference. Similarly, for *RIP-LCMV-GP* mice, data from our colony over the last year show that 100% of mice will develop diabetes. Therefore, assuming that 99% of *RIP-LCMV-GP* mice will develop diabetes if left untreated and that TYK2 inhibitor treatment will reduce the probability of disease by 80% (i.e. down to 19.8%), at a power of 0.80, an alpha of 0.05, and using a 1-sided fishers exact conditional test, a minimum of 7 mice are needed in each group (*RIP-LCMV-GP* and TYK2-treated mice) to detect this difference.

The quantitative polymerase chain reaction (qPCR) and Western blot analyses were conducted on datasets comprising 3–6 biological replicates. A Shapiro Wilks test was used to assess normality, and an F-test for homogeneity of variance was performed between the groups if the data was normally distributed. If the data did not assume normality, a non-parametric statistical test was used to determine the differences. A one-way analysis of variance (ANOVA) was used for multiple comparisons. An ordinary one-way ANOVA with Sidak's post hoc test was used to compare the data from gene expression studies across drug dosage groups. The Log-rank (Mantel–Cox) test was used to determine differences in diabetes development. For the *RIP-LCMV-GP* model, the time of LCMV administration (day 0) was considered to be the origin, and for the survival analysis, day 11 (where we first start to observe diabetes incidence) was considered the start and was continued until the experimental endpoint (14 days post-inoculation). For NOD studies, six weeks of age was considered to be the origin (initiation of drug administration), and for survival analysis, week 14 was considered to be the starting point and was continued until the experimental endpoint (25-weeks of age). A Mann–Whitney U test was used to evaluate mRNA, protein expression, and insulitis scoring to determine statistical differences between vehicle- and TYK2i-treated mice. All data analyses were performed utilising GraphPad Prism, version 10. The statistical analysis for spatial transcriptomics and proteomics data are discussed above in their respective methods section.

### Role of funders

The funding sources did not play a role in study design, data collection, data analyses, interpretation, or writing of the report.

## Results

### TYK2 inhibitors repress IFNα signalling and inflammatory gene expression in human β cells

**Fig. 1:**
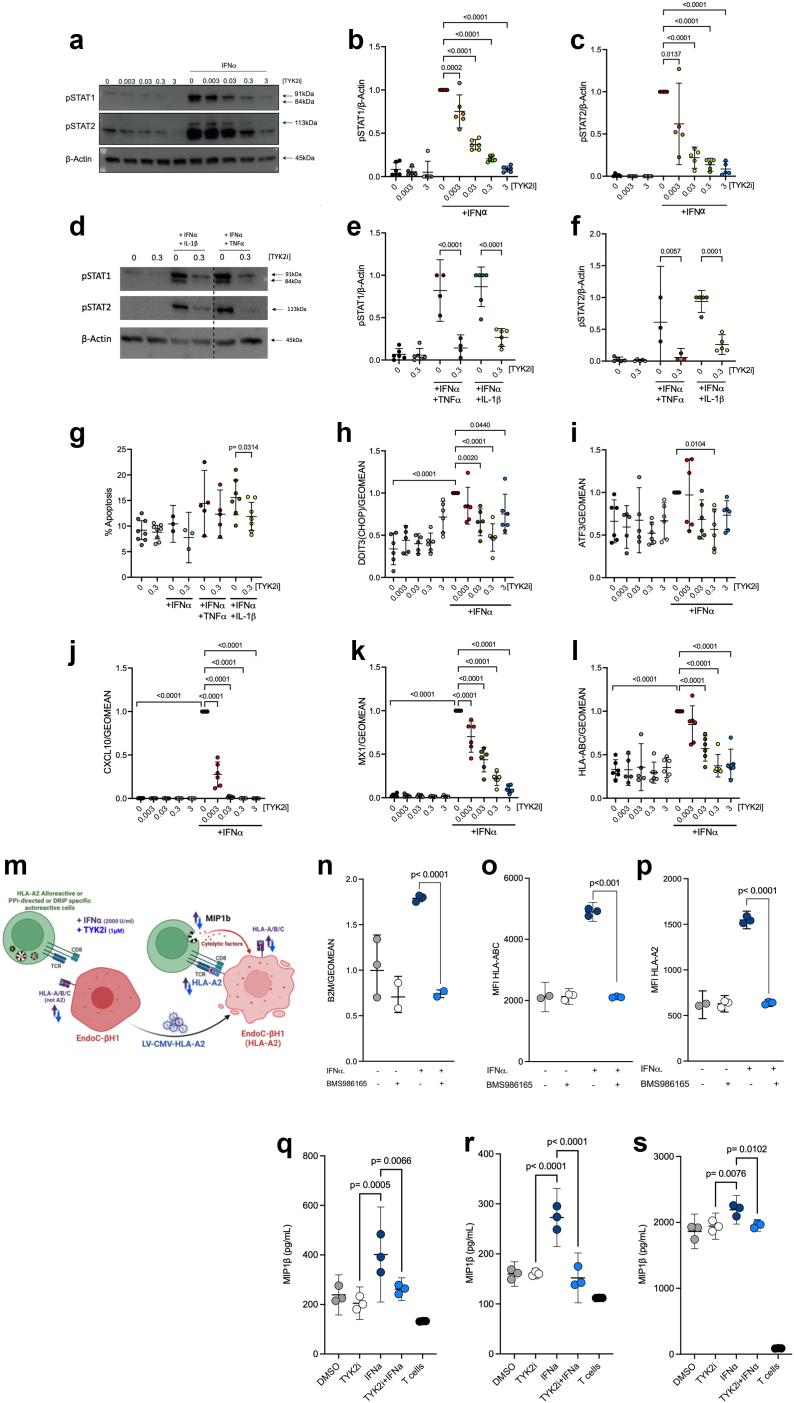
**TYK2 inhibitors repress IFNα signalling and inflammatory gene expression and reduce β cell immunogenicity**. Dispersed human islet cells were allowed to rest in culture for two days before pre-treatment for 2 h with the indicated concentrations (expressed in mM) of TYK2i BMS-986165. Treatment was continued with BMS-986165 in the absence or presence of IFNα (2000 U/mL), IFNα (2000 U/mL) + TNFα (1000 U/ml), or IFNα (2000 U/mL) + IL-1β (50 U/mL) for 24 h. (**a–f**) Western blotting of protein lysates was performed to detect phospho-STAT1/2. Data were normalised to the levels of β-actin and expressed relative to treatment with IFNα alone. (**g**) Apoptotic cells were identified by Hoechst 33342 and propidium iodide staining. (**h–l**) Total RNA was extracted and mRNA levels of (**h**) *DDIT3* (*CHOP*), (**i**) *ATF3*, (**j**) *CXCL10*, (**k**) *MX1*, and (**l**) *HLA-ABC* were analysed by RT-qPCR. (**m**) Schematic representation of the experimental workflow on EndoC-βH1/HLA-A2 cells co-cultured either with HLA-A2 alloreactive or autoreactive CD8+ T cell clones directed against insulin defective ribosomal product and/or preproinsulin specific T cells. (**n**) RT-qPCR analysis showing *B2M* expression in EndoC-BH1/HLA-A2 cells upon IFNα treatment (2000 U/mL) in the presence or absence of 1 uM BMS-986165. (**o**) Surface expression of HLA-ABC and (**p**) HLA-A2 in EndoC-βH1/HLA-A2 cells prior to co-culture. Cells were exposed to IFNɑ in the presence or absence of BMS-986165 for 24 h. T cell activation assay (MIP-1β release) after co-culture with **(q)** A2 alloreactive T cells, (**r)** autoreactive T cells directed against insulin defective ribosomal product, or **(s)** preproinsulin specific T cells. For RT-qPCR analysis, expression data were normalised by the geometric mean of *GAPDH* and *ACTB* expression levels and expressed relative to cells exposed to IFNα alone. Statistical analyses for human islet studies (n = 3–7) were conducted using one-way ANOVA with multiple comparisons between treatment groups. Co-culture experiments were performed with independent biological replicates (n = 3). An unpaired t-test (with or without Welch's correction) was applied to evaluate the expression of surface proteins (panels n, o, and p), while one-way ANOVA with multiple comparisons was used to analyse differences between treatment conditions (all remaining panels excluding a, d, and m). All data are presented as the mean with 95% confidence intervals (CI).

To investigate the direct effects of TYK2i treatment on IFNα responses in β cells, islets isolated from human organ donors were treated with IFNα in the presence or absence of BMS-986165 (deucravacitinib), which is FDA-approved for use in psoriasis.^33^ As anticipated, IFNα treatment stimulated phosphorylation of STAT1 and 2, (P < 0.0001, one-way ANOVA) and this early effect was repressed by the TYK2i in a dose-dependent manner (Fig. 1a–c). Additionally, co-incubation of human islets with TYK2i (0.3 μM) and combinations of proinflammatory cytokines (IFNɑ+TNFɑ or IFNɑ+IL-1β) prevented the activation of pSTAT1and pSTAT2 (P < 0.0001, one-way ANOVA) (Fig. 1d–f). TYK2i also reduced cytokine-induced apoptosis (Fig. 1g) and decreased the expression of genes involved in ER stress (*DDIT3 (CHOP)* and *ATF3*) (Fig. 1h and i). Furthermore, the increased expression of a selected signature of IFNα-responsive genes involved in chemokine production (*CXCL10*) (P < 0.0001, one-way ANOVA), antiviral responses (*MX1*) (P < 0.0001, one-way ANOVA), and antigen presentation that contributes to CD8+ T cell target recognition (*HLA-ABC*) (P < 0.0001, one-way ANOVA) was blocked effectively by TYK2i (Fig. 1j-l). Of note, we have previously shown that TYK2i-induced downregulation of *MX1* does not increase the susceptibility of human β cells to infections by the potentially diabetogenic coxsackievirus B (CVB).^70^

Next, we performed side-by-side comparisons between BMS-986202 and BMS-986165 using preparations of human EndoC-BH1 β cells. The two TYK2is were highly potent, efficacious, and largely similar in their capacity to decrease the expression of IFNα-responsive genes (Supplemental Figure S1). TYK2 inhibition also repressed the increase in inflammatory gene expression in response to co-incubation with IFNα+TNFα or IFNα+IL-1β and under conditions of extended IFNα incubation up to 48 h (Supplemental Figure S2). Notably, the inhibitors did not cause cellular toxicity, as treatment with BMS-986202 or BMS-986165 alone did not increase the percentage of basal apoptosis (Fig. 1g).

Next, we evaluated the potential for BMS-986202 to modulate the response to IFNα in newly differentiated β cells derived from human inducible pluripotent stem cells (iPSCs). iPSC-derived islet cells are not fully differentiated and provide an interesting model for human islets in the first months of life, a period when β cell autoimmunity may begin in some cases.^20^ Differentiation of HEL115.6 iPSC cells into endocrine pancreatic cells was performed with quality control evaluations at stages 1, 4, and at end-stage (stage 7), as previously described.^45^^,^^71^ Dispersed islet-like cell aggregates were preincubated in the presence or absence of TYK2i, followed by co-incubation with the inhibitor and IFNα alone or in combination with TNFα or IL-1β for up to 48 h. Similar to the results obtained with adult human islet preparations and EndoC-βH1 cells, TYK2i blocked the cytokine-induced increase in the expression of *CXCL10, MX1,* and *HLA-ABC* (Supplemental Figure S2g–l), and there was a trend towards decreased apoptosis in iPSCs treated with TYK2i (Supplemental Figure S2m). TYK2i was unable to prevent upregulation of ER-stress transcripts induced by classical chemical ER stressors (brefeldin A or thapsigargin), suggesting that IFNα may mediate ER stress through non-canonical pathways. Consistent with this notion, neither brefeldin A or thapsigargin increased levels of total or pSTAT (Supplemental Figures S3a–f and S4a–b).

### TYK2 inhibitors do not alter the expression of β cell identity genes

A recent publication showed that TYK2 knockout in human iPSCs delayed endocrine cell differentiation without impacting mature stem cell-derived islet function.^34^ Therefore, we tested the impact of BMS-986202 on the expression of β cell identity genes in adult human islets. Importantly, TYK2i treatment did not alter the expression of mRNAs encoding either insulin or PDX1 (Supplemental Figure S5a and b).

### TYK2 inhibitors reduce β cell immunogenicity

We have shown that TYK2 inhibition blocks the transcriptional effects of IFNα on β cells; however, it remains to be clarified whether blocking these responses influences interactions, either positive or negative, between β cells and T lymphocytes in the context of insulitis. Among the diverse effects of IFNα, this cytokine induces expression of the checkpoint inhibitor *PD-L1* in β cells. Indeed, we observed that *CD274 (PD-L1)* mRNA expression in human islets was increased by IFNα treatment but repressed by TYK2 inhibition (Supplemental Figure S5c). This finding raises the possibility that TYK inhibition could have an untoward effect of heightening deleterious β and T cell interactions, in spite of the parallel decrease in CXCL10 and HLA class I expression induced by TYK2is (Fig. 1j and l). To test this possibility, we used several CD8 T cell clones (e.g. HLA-A2 alloreactive and autoreactive clones directed against preproinsulin and an insulin defective ribosomal product) and evaluated the effect of the TYK2i on antigenic peptide presentation to T cells (Fig. 1m).^51^, ^52^, ^53^^,^^72^^,^^73^ EndoC-βH1 cells expressing HLA-A2 were generated by transduction with a lentiviral vector containing HLA-A02:01.^74^ Upon IFNα stimulation, we observed an increase in *B2M* gene expression (IFNα vs IFNα + TYK2i, P < 0.0001, unpaired t-test) (Fig. 1n) that correlated with increased in HLA-A/B/C (IFNα vs IFNα + TYK2i, P < 0.001, unpaired t-test with Welch's correction) and HLA-A2 surface expression (IFNα vs IFNα + TYK2i, P < 0.0001, unpaired t-test) (Fig. 1o,p), as reported previously.^75^ This effect was effectively prevented by co-incubation with BMS-986165 (Fig. 1n). In agreement with these findings, IFNα increased peptide specific T cell activation, as monitored by MIP-1β production and release, and this effect was reduced by co-treatment with BMS986165 in all conditions tested (Fig. 1q. IFNα vs TYK2i, P = 0.0005 and IFNα vs IFNα+TYK2i, P = 0.0066; Fig. 1r. IFNα vs TYK2i, P < 0.0001 and IFNα vs IFNα+TYK2i, P < 0.0001; Fig. 1s. IFNα vs TYK2i, P = 0.0076 and IFNα vs IFNα+TYK2i, P = 0.0102; one-way ANOVA) (Fig. 1q–s). Therefore, the overall effect of TYK2i on β cell immunogenicity was beneficial, limiting antigenic peptide presentation during inflammation.

Collectively, these *in vitro* results confirm that TYK2i treatment is sufficient to: 1) block the deleterious IFNα-mediated transcriptional effects in isolated human islets, EndoC-βH1 cells, and iPSC-derived β cells without modifying the differentiated β cell phenotype and 2) decrease the susceptibility of β cells to effector T cell attack.

### TYK2i decreases T1D development in the *RIP-LCMV-GP* mouse model

**Fig. 2:**
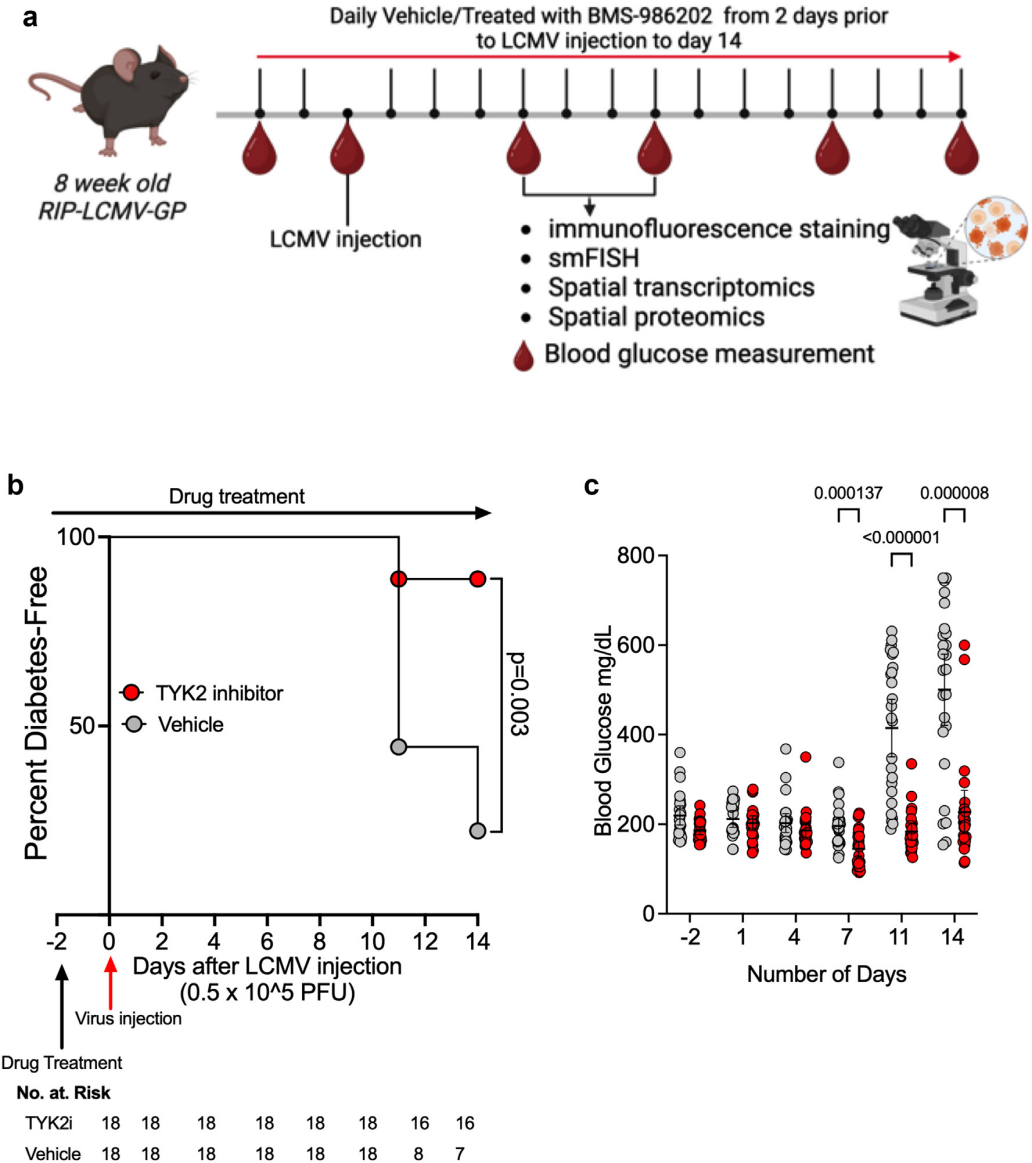
**TYK2 inhibition reduces the onset of diabetes in the *RIP-LCMV-GP* mouse model of T1D**. (**a**) Male *RIP-LCMV-GP* mice were inoculated at 8 weeks of age with LCMV stock (0.5 × 10^5^ PFU I.P., Armstrong strain), and blood glucose levels were measured pre-inoculation (Pre) and on days 1, 4, 7, 11, and 14 post-inoculation. Mice were pre-treated with either vehicle or TYK2i BMS-986202 (30 mg/kg/day) for 2 days prior to inoculation, and treatment continued until the study end on day 14. n = 18 mice/group. Vehicle- and TYK2i-treated groups are shown in grey and red symbols or bars, respectively. (**b**) Kaplan–Meier diabetes incidence plot for vehicle- and TYK2i-treated groups. (**c**) Blood glucose levels of vehicle- and TYK2i-treated mice. All blood glucose data are presented as mean with 95% CI, with individual data points represented: significant differences determined by Log-rank (Mantel–Cox) test.

BMS-986202 was selected for *in vivo* studies based on our *in vitro* data (see above) and the compound's mouse-optimised pharmacokinetic properties.^37^ First, we tested the impact of BMS-986202 in *RIP-LCMV-GP* mice, a model created by placing the glycoprotein (GP) of the lymphocytic choriomeningitis virus (LCMV) under transcriptional control of the rat insulin promoter (RIP). Upon LCMV inoculation, *RIP-LCMV-GP* mice develop diabetes within 14 days, and disease progression mimics elements of human disease, including dependence upon IFNα signalling and induction of memory CTLs that elicits effector T cell-mediated destruction of β cells.^26^ In this first series of *in vivo* experiments, 8-week-old *RIP-LCMV-GP* mice were pretreated with vehicle or BMS-986202 (30 mg/kg/day) for 2 days and then inoculated with LCMV (0.5 × 10e5 PFU I.P; Armstrong strain) to induce the onset of T1D. Drug or vehicle treatment was continued for 14 days post-LCMV inoculation, and blood glucose levels were measured before LCMV inoculation and on days 1, 4, 7, 11, and 14 post-inoculation (Fig. 2a). The criterion for established diabetes was two consecutive blood glucose readings exceeding 250 mg/dL.

Treatment of *RIP-LCMV-GP* mice with BMS-986202 significantly reduced the incidence of diabetes: 89% of BMS-986202-treated mice remained diabetes-free throughout the duration of the study period, while only 17% of the mice in the vehicle-treated group remained diabetes-free (P = 0.003, Hazard Ratio: 0.18; 95% Confidence Interval (CI): 0.06–0.53, Log-rank (Mantel–Cox) test, Fig. 2b).^76^ Consistent with the observed protection from diabetes incidence, TYK2i-treated mice had lower blood glucose levels on days 11 and 14 post-inoculation compared to vehicle-treated mice vehicle vs TYK2i, day 7 (P = 0.00013), day 11 (P < 0.0001), day 14 (P < 0.0001), Multiple Mann–Whitney test (Fig. 2c).

**Fig. 3:**
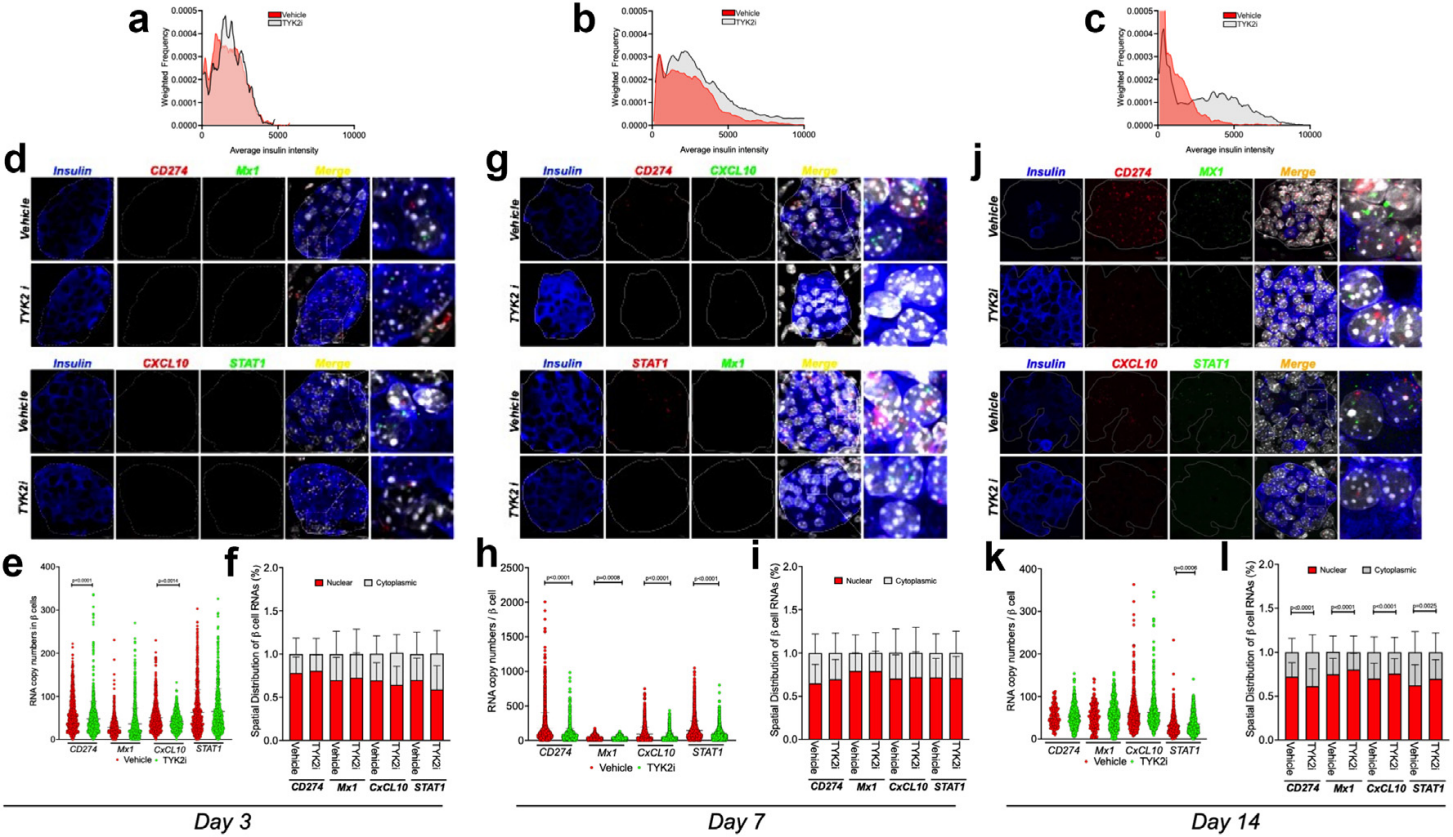
**TYK2i treatment preserves pancreatic β cells and inhibits the expression of IFNα-induced mRNAs in *RIP-LCMV-GP* mice**. Pancreas tissue was harvested from vehicle- and TYK2i-treated *RIP-LCMV-GP* mice on days 3, 7, and 14 post-inoculation. Single-molecule fluorescence *in situ* hybridisation (smFISH) detecting *Cd274*, *Mx1, Cxcl10*, and *Stat1* was performed in combination with co-staining of insulin protein and DAPI for nuclear labelling. mRNA expression in individual insulin-positive β cells was quantified following an established pipeline (details are provided in Methods). (**a–c**) Histogram showing the cellular insulin intensity from the pancreatic tissue sections of vehicle (red plot lines) and TYK2i-treated mice (grey plot lines) on days 3, 7, and 14. **(d**–**e)** Representative smFISH images of *Cd274*, *Mx1*, *Cxcl10*, and *Stat1* in mouse pancreatic islets on day 3 **(d)**, 7 (**g**), and 14 (**j**). Islet regions are delineated, and the merged smFISH images are shown along with a designated square region of expanded magnification (far right images). (**e, h, k**) Quantitation of mRNA copy number in a single β cell of a pancreatic islet on days 3, 7, and 14. (**f, i, l**) Spatial analysis of RNA localisation between nuclear and cytoplasmic subcellular compartments. Individual β cell measurements of RNA copies and RNA localisation data are expressed as mean ± SD with an indication of significant differences, n = 5–7 mice were studied per condition and 4–10 islets were randomly selected from each section; statistical significance was determined by a Mann–Whitney U test.

To determine the impact of *in vivo* TYK2 inhibition on β cells, we performed single-molecule fluorescence *in situ* hybridisation (smFISH) for a representative set of IFNα-induced mRNAs (*Cd274, Cxcl10, Stat1,* and *Mx1*), and we quantitated protein expression for IFN-target genes using immunofluorescence. For smFISH, we co-stained for insulin protein to allow for β cell segmentation in pancreatic tissue sections collected from *RIP-LCMV-GP* mice on days 3, 7, and 14 post-LCMV inoculation (Fig. 3). On day 3, there was no difference in insulin protein expression between vehicle- and BMS-986202-treated mice (Fig. 3a); however, a higher insulin intensity was observed in islets of TYK2i-treated mice on days 7 and 14 (Fig. 3b and c). Quantification of IFNα-induced mRNAs in β cells *in situ* revealed a significant decrease in *Cd274* (P < 0.0001, Mann–Whitney test) and *Cxcl10* expression (P < 0.0014, Mann–Whitney test) (Fig. 3d and e). In contrast, we observed an increase in *Mx1* and *Stat1* mRNA expression in TYK2i-treated mice at day 3 post-LCMV inoculation (Fig. 3e). At day 7, there was a significant decrease in all four mRNAs in β cells of the TYK2i-treated mice (*CD274*, P < 0.0001; *Mx1*, P = 0.0008; *CxCL10*, P < 0.0001, and *Stat1*, P < 0.0001, Mann–Whitney test) (Fig. 3g and h), and at day 14, a persistent reduction in *Stat1* mRNA (*Stat1*, P = 0.0006, Mann–Whitney test) was observed in TYK2i-treated mice (Fig. 3j and k). Because the spatial localisation of RNAs represents an important post-transcriptional mechanism that patterns gene expression,^77^, ^78^, ^79^ we quantitated the cytoplasmic and nuclear distribution of mRNA. This analysis revealed no significant differences in the spatial localisation (cytoplasmic vs nuclear) of the mRNAs on day 3 (Fig. 3f) or day 7 (Fig. 3I). However, at day 14 post-LCMV inoculation, *Cd274* (Vehicle vs TYK2i, P < 0.0001, Mann–Whitney test) exhibited increased cytoplasmic localisation, while *Mx1*, *Cxcl10,* (P < 0.0001) and *Stat1* (P < 0.0025, Mann–Whitney test) showed increased nuclear localisation in TYK2i-treated mice (Fig. 3l), suggesting that changes in the spatial distribution of these RNAs might play a role during the evolution of cellular stress and in response to TYK2 inhibition.

To understand how these changes in mRNA levels and spatial localisation affected protein expression patterns, immunofluorescence staining of PD-L1, CXCL10, and insulin was performed in pancreatic tissue sections at days 3, 7, and 14 post-LCMV inoculation (Supplemental Figure S5d and e). Insulin levels in islets from TYK2i-treated mice remained stable throughout the study time points. In contrast, vehicle-treated mice exhibited a transient increase in insulin expression (P < 0.0001, Mann–Whitney test) on day 3 (Supplemental Figure S5e), which gradually declined by days 7 and 14 (Supplemental Figure S5e). Consistent with our smFISH analysis, PD-L1 (P < 0.0001, Mann–Whitney test) and CXCL10 (P < 0.0001, Mann–Whitney test) protein levels were reduced in β cells of TYK2i-treated mice on both days 3 and 7, indicating early modulation of IFNα induced genes *in vivo* by TYK2i treatment (Supplemental Figure S5e).

### TYK2 inhibition decreases early innate immune responses in the whole blood and pancreatic lymph nodes of *RIP-LCMV-GP* mice

**Fig. 4:**
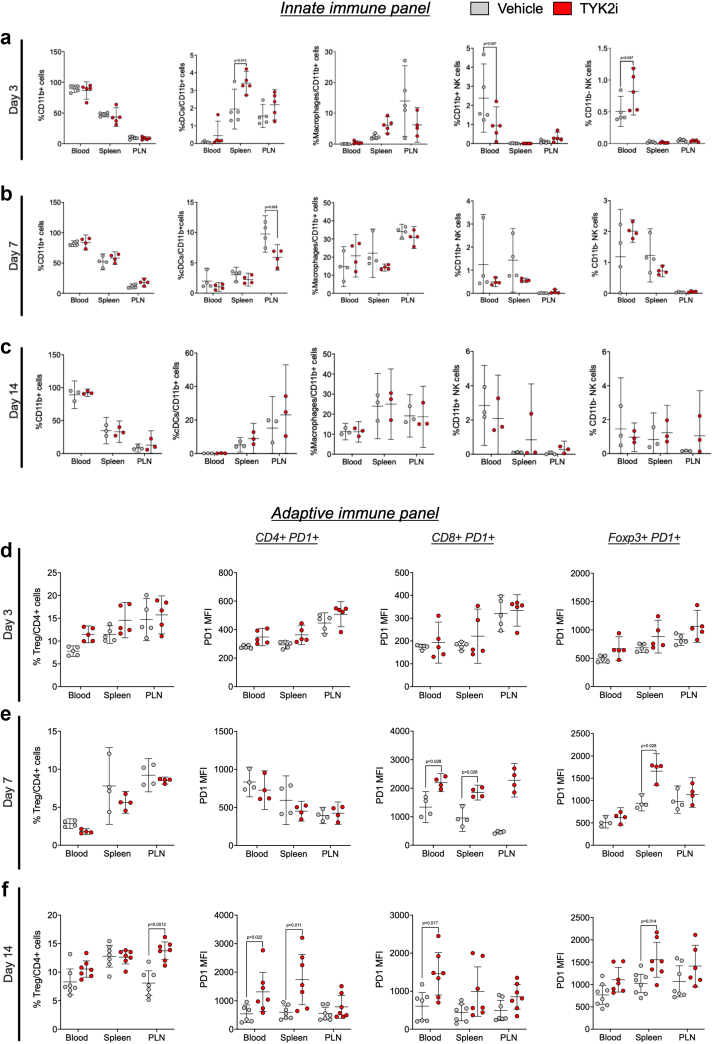
**TYK2 inhibition reshapes innate and adaptive immunity in blood, spleen, and PLN of RIP-LCMV-GP mice**. Peripheral blood mononuclear cells (PBMCs), splenocytes, and immune cells from PLN were isolated and labelled with CD11b^+^MHCII^+^CD11c^+^ (dendritic cells, DCs), CD11b^+^MHCII^+^F4/80^+^ (macrophages), CD11b^+^CD49^+^ (mature NK cells), and CD11b^+^CD49^-^ (immature NK cells). Immune cell characterisation was performed using flow cytometry. **(a**–**c)** Percentage of DCs, macrophages, mature NK cells, and immature NK cells from 3, 7, and 14-days post-inoculation from vehicle (grey) and TYK2i-treated (red) *RIP-LCMV-GP* mice. (**d–f**) Percentage of CD4^+^FoxP3^+^CD25^+^ Tregs, CD4^+^PD1^+^ T cells, and CD8^+^PD1^+^ T cells from 3, 7, and 14-days post-inoculation from vehicle (grey) and TYK2i-treated (red) *RIP-LCMV-GP* mice. Data are presented as mean with 95% CI, and individual data points are included, n = 3–5 mice per condition; Statistical significance was determined by a Mann–Whitney U test.

To determine if the protective effect of TYK2 inhibition on T1D development in *RIP-LCMV-GP* mice was associated with altered innate or adaptive immune responses, flow cytometry analysis was performed on the whole blood, spleen, and PLN of vehicle- and TYK2i-treated mice at days 3, 7, and 14 post-LCMV inoculation. The gating strategy for the identification of innate immune cell populations is provided in Supplemental Figure S6a. Treatment with TYK2i did not alter the percentage of CD11b^+^ cells in the blood, spleen, or PLN, indicating that the myeloid cell population assayed was largely unaffected (Fig. 4a–c). However, early in the disease progression (day 3), there was an increase in the percentage of dendritic cells (DCs, CD11b^+^/CD11c^+^/MHCII^+^) in the spleen (P = 0.015, Mann–Whitney test) and a trend towards decreased percentages of proinflammatory macrophages (F4/80^+^/CD11b^+^/MHCII^+^) in the PLN of TYK2i-treated mice. Furthermore, BMS-986202 treatment decreased the number of mature natural killer (NK) cells (CD49b^+^/CD11b^+^) (P = 0.047) and increased the subset of immature NK cells (CD49b^+^/CD11b^−^) in the blood (P = 0.047, Mann–Whitney test) (Fig. 4a). At day 7, a critical time point preceding the onset of hyperglycemia in vehicle-treated mice, there was a lower percentage of DCs (CD11c^+^/MHCII^+^) in the PLN of TYK2i-treated mice (P = 0.028, Mann–Whitney test) (Fig. 4b). These findings indicate that TYK2 inhibition decreases activation of the early innate immune responses in the blood and PLN in this murine model of viral-induced T1D.

### TYK2 inhibition alters the adaptive immune repertoire in the whole blood, spleen, and PLN of *RIP-LCMV-GP* mice

To investigate the impact of TYK2i on adaptive immune responses, we analysed whole blood, spleen, and PLN on day 3, 7, and 14 post-LCMV inoculation using flow cytometry with the gating strategy described in Supplemental Figure S6b. This analysis revealed distinct changes in T cell subsets following TYK2 inhibition. However, in contrast to effects on innate immune responses, these effects were observed at the later time points (Fig. 4d–f). At day 7 post-inoculation, CD8+ T cells from TYK2i-treated mice showed a striking upregulation of PD-1, the receptor of PD-L1, in the blood (P = 0.028), spleen (P = 0.028), and PLN (P < 0.0001, Mann–Whitney test) (Fig. 4e). This upregulation continued through day 14 in the blood and spleen (Fig. 4f). At day 14, there was also an increase in the percentage of CD4+PD1^+^ T cells in the blood and spleen of TYK2i-treated mice. Additionally, immune-suppressive regulatory T cells (Tregs, CD25+/FoxP3+) positive for PD-1 (CD25+Foxp3+PD1+) were increased in the spleen of TYK2i-treated mice at days 7 (P = 0.028) and 14 (P = 0.014). Moreover, at the 14-day time point, there was a parallel increase in the percentage of Tregs in the PLN (P = 0.0012) (Fig. 4f). These results suggest that TYK2 inhibition leads to upregulation of PD-1 on CD4+ (blood, P = 0.022; spleen, P = 0.011), CD8+ (blood, P = 0.017), and FoxP3+ cells (spleen, P = 0.014) in a time-dependent manner in *RIP-LCMV-GP* mice.

### TYK2 inhibition attenuates the development of T1D in NOD mice

**Fig. 5:**
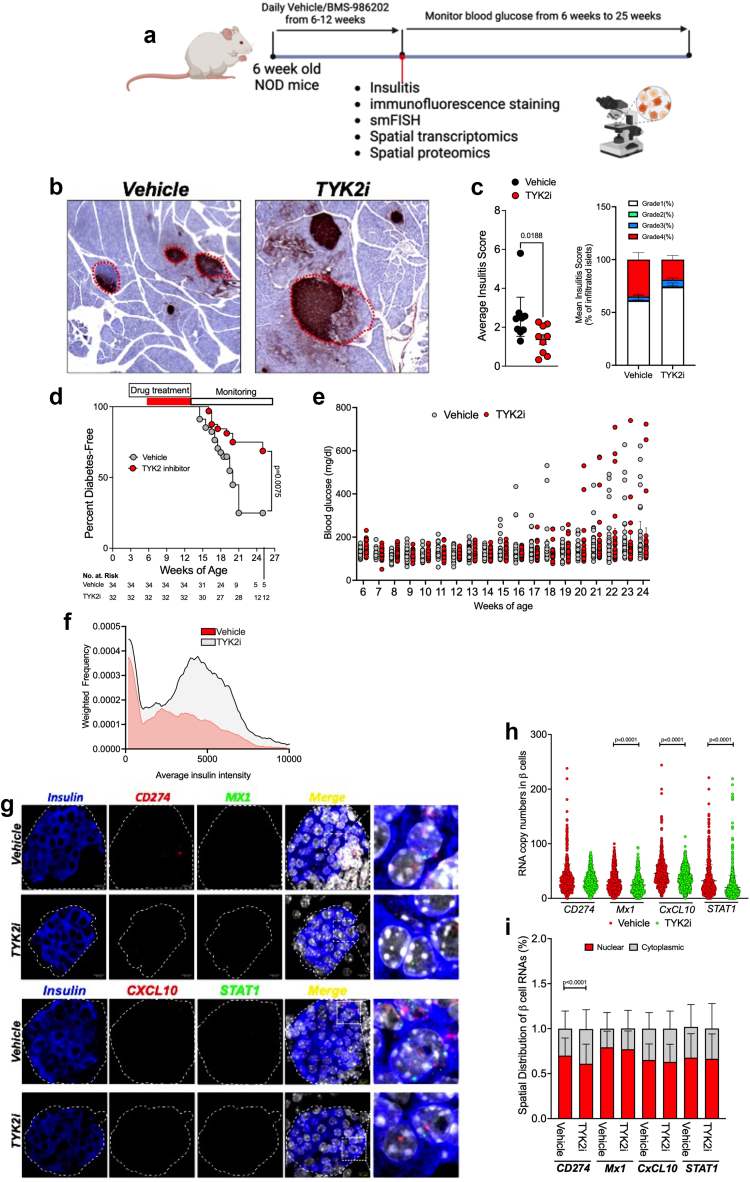
**TYK2 inhibition mitigates diabetes onset and IFNɑ responses in female NOD mice**. (**a**) Six-week-old female NOD mice were treated with vehicle or TYK2i (BMS986202, 30 mg/kg) by oral gavage once daily for six weeks and monitored for diabetes onset (n = 34 vehicle/32 TYK2i). A separate cohort of NOD mice (n = 9 per study condition) was sacrificed at the end of TYK2i dosing (12 weeks of age) for tissue analysis. Blood glucose levels were measured weekly from 6 weeks until diabetes conversion (14 weeks) and biweekly until the end of the study time point. Diabetes was defined as blood glucose levels of ≥250 mg/dL in two consecutive measurements. (**b–c**) Representative images of insulin immunohistochemistry and insulitis scoring (mean with 95% CI). The stacked bars represent the percentage of different grades of immune cell infiltration across vehicle- and TYK2i-treated NOD mice. (**d**) Kaplan–Meier diabetes incidence plot. (**e**) Blood glucose levels. Vehicle-treated mice are indicated in grey bars and TYK2i-treated mice are represented by red bars. (**f**) Quantification of insulin expression in pancreatic β cells. (**g**) Representative images of smFISH for *Cd274*, *Cxcl10*, *Stat1*, and *Mx1*. Islet regions are delineated, and the merged smFISH images are included along with a designated square region of expanded magnification (far right images). (**h**) Quantitation of mRNA copy numbers of *Cd274*, *Cxcl10*, *Stat1*, and *Mx1* in β cells. (**i**) Spatial analysis of RNA localisation between nuclear and cytoplasmic cellular compartments. For diabetes incidence studies, statistical analysis was performed by Log-rank (Mantel–Cox) test for the Kaplan–Meier plot. Wilcoxon rank sum tests were used to determine the effect of TYK2 inhibition on immune cell infiltration presented as an insulitis score. For smFISH, individual β cell measurements of RNA copy are presented, RNA localisation data are expressed as mean ± SD, and the Mann–Whitney U test was used to determine statistical differences.

To investigate the impact of TYK2 inhibition in a spontaneous preclinical model of T1D, female nonobese diabetic (NOD) mice were treated with either vehicle or 30 mg/kg BMS-986202 for 6 weeks beginning at 6 weeks of age. In this model, the spontaneous conversion of female NOD mice to diabetes at approximately 14 weeks of age reflects the onset of insulitis and T cell-mediated destruction of β cells with a high dependence upon IFNα.^25^^,^^56^ Animals treated with vehicle or TYK2i were monitored for blood glucose levels once a week from 6 to 14 weeks of age and biweekly from 15 to 24 weeks of age (Fig. 5a). The criteria for the development of diabetes was two consecutive blood glucose readings that exceeded 250 mg/dL. Pancreatic tissue from vehicle- and BMS-986202-treated mice was collected from a cohort of mice at the end of treatment (12 weeks of age) for histological evaluation of immune cell infiltration into islets. Perivascular/periductal immune cell infiltration and average insulitis score was reduced in the pancreas of mice treated with BMS-986202 compared to vehicle-treated mice (P = 0.0188, Mann–Whitney test) (Fig. 5b and c). By 24 weeks of age, the incidence of diabetes-free mice within the vehicle cohort was less than 25%, whereas 70% of the mice receiving BMS-986202 remained diabetes-free (P = 0.0075, Log-rank (Mantel–Cox) test (Fig. 5d) with well-maintained glycaemia (Hazard ratio: 0.35; 95% Confidence Interval: 0.16–0.74, Fig. 5e).^76^

Similar to the analysis performed in the *RIP-LCMV-GP* mice, smFISH was performed on the pancreas from 12-week-old vehicle- and TYK2i-treated NOD mice to detect IFNα-mediated inflammatory gene expression in β cells (Fig. 5f–i). Overall, the signature of inflammatory gene expression was reduced in β cells from TYK2i-treated mice, with significantly lower levels of *Mx-1* (P < 0.0001), *CxcL-10* (P < 0.0001), and *Stat1* mRNAs (P < 0.0001) (Fig. 5g–h), while the cytoplasmic localisation of *Cd2*74 mRNA (P < 0.0001, Mann–Whitney U test) was significantly increased (Fig. 5i). Immunofluorescence staining of pancreatic tissue sections for PD-L1, CXCL10, and insulin did not reveal significant differences between the groups at 12 weeks of age (Fig. 5f and Supplemental Figure S7a and b).

### Evaluation of spatial dynamics reveals that TYK2 inhibition reshapes inflammatory signalling and cellular composition in T1D models

**Fig. 6:**
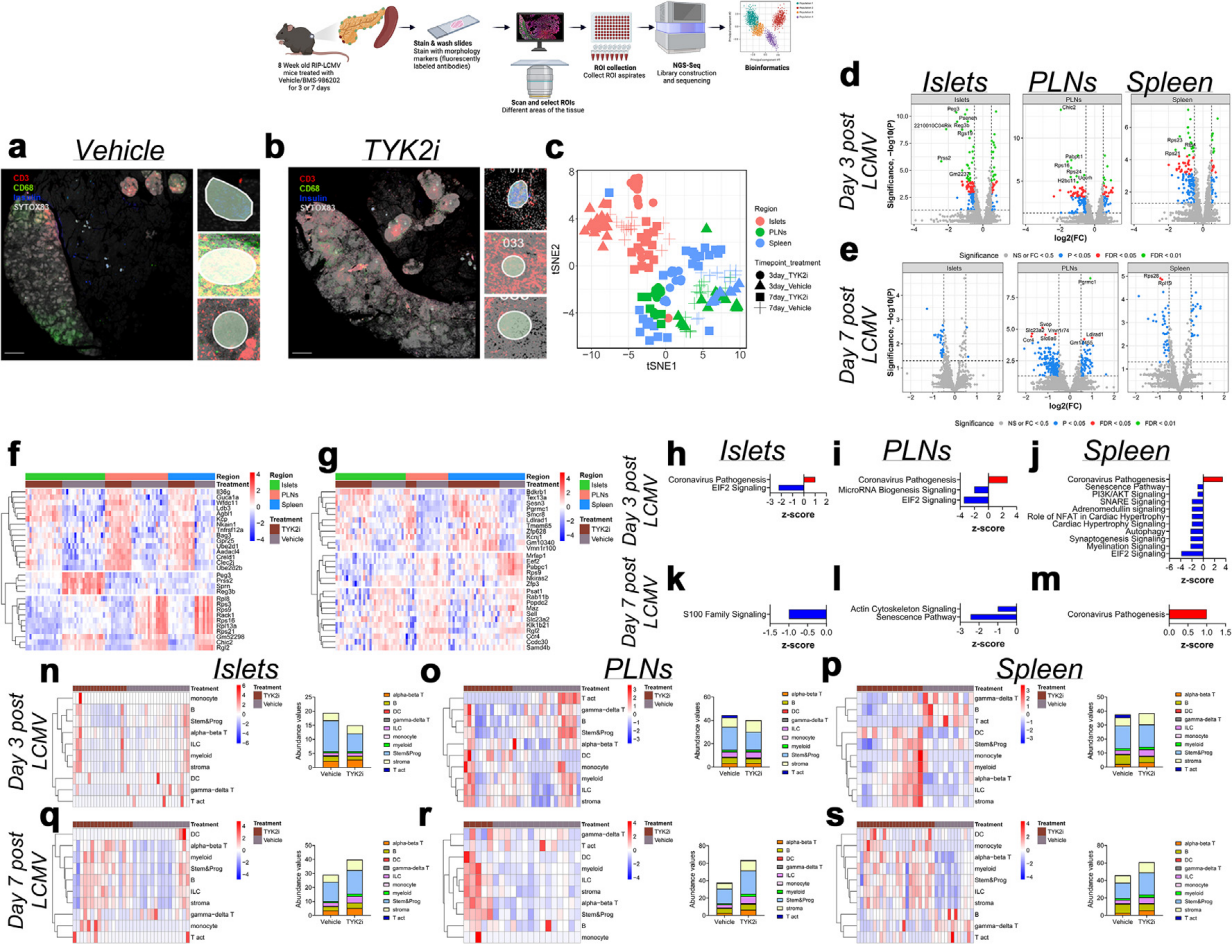
**TYK2 inhibition alters spatial transcriptome profiles in islets, PLN, and spleen of *RIP-LCMV-GP* mice**. Tissues were harvested from vehicle- and TYK2i-treated *RIP-LCMV-GP* mice on days 3 and 7 post-inoculation. (**a–b**) Representative images of the islets, PLN, and spleen stained for CD3 (red), CD68 (green), insulin (blue), and Sytox83 (grey) nuclear staining. (**c**) tSNE scatter plot of sample clustering between vehicle- and TYK2i-treated mice at days 3 and 7 post-inoculation in the islets, PLN, and spleen. (**d–e**) Volcano plots of differentially expressed genes between vehicle- and TYK2i-treated mice at (**d**) day 3 and (**e**) day 7 post-inoculation. (**f–g**) Top 10 differentially expressed genes from each selected ROI of vehicle- and TYK2i-treated mice at (**f**) day 3 and (**g**) day 7 post-inoculation. (**h–m**) Ingenuity pathway analysis showing upregulated (red bar) and downregulated (blue bar) pathways in islets, PLN, and spleen at (**h–j**) day 3 and (**k–m**) day 7 post-inoculation. (**n–s**) Deconvolution analysis of immune cells showing the abundance of different immune cell populations from islets, PLN, and spleen at (**n–p**) day 3 and (**q–s**) day 7 post-inoculation. n = 3–4 mice per group. The data in i and j are presented as mean ± SD. A Mann–Whitney U test was used to identify statistical differences.

**Fig. 7:**
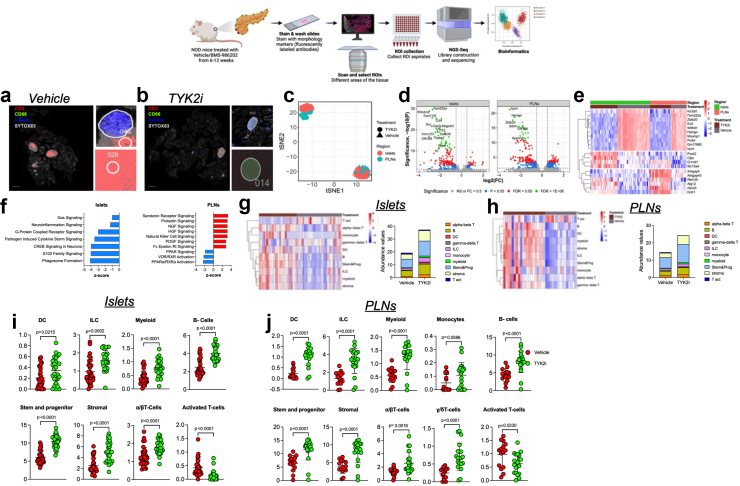
**Spatial whole transcriptome analysis reveals diminished inflammatory gene expression in TYK2i-treated islets and PLN of NOD mice**. Islets and PLNs were harvested from vehicle- and TYK2i-treated NOD mice at 12 weeks of age. (**a–b**) Representative images of islets and PLN stained for CD3 (red), CD68 (green), insulin (blue), and Sytox83 (grey; nuclear staining). (**c**) tSNE scatter plot showing clustering of samples from islets and PLN between vehicle- and TYK2i-treated 12-week-old female NOD mice. (**d**) Volcano plots showing differentially expressed genes from islets and PLN between vehicle- and TYK2i-treated mice. (**e**) Top 10 differentially expressed genes from each selected ROI in islets and PLN of mice treated with vehicle or TYK2i. (**f**) Ingenuity pathway analysis showing upregulated (red bars) and downregulated (blue bars) pathways in islets and PLN of NOD mice. (**g–j**) Deconvolution analysis of immune cells showing the abundance of different immune cell populations from (**g**) islets and (**h**) PLN from NOD mice treated with vehicle or TYK2i; abundance of immune cell populations showing significant differences between vehicle- and TYK2i-treated NOD mice from (**i**) islets and (**j**) PLN. n = 3–4 mice/group. Dotplot showing the proportion of cell types present in each ROI identified by spatial deconvolution. A Mann–Whitney U test was used to compare the statistical significance between vehicle- and TYK2i-treated groups.

We reasoned that an in-depth examination of the impact of TYK2 inhibition would allow us to compare responses across the immune and endocrine compartments, while potentially providing additional insight into their effects on disease pathogenesis. Therefore, we used a GeoMx spatial whole transcriptomic atlas (WTA) to evaluate islets, spleen, and PLN from *RIP-LCMV-GP* mice at days 3 and 7 post-inoculation (Fig. 6a–s and Supplemental Figure S8) and islets and PLN from NOD mice treated with vehicle or TYK2i (Fig. 7a–j and Supplemental Figure S9). GeoMx WTA probes were hybridised, and the tissue sections were stained for insulin, CD3, and CD68 to select ROIs (Fig. 6a–b and Fig. 7a–b). A total of 193 ROIs were selected from *RIP-LCMV-GP* mice, and 92 ROIs were selected from NOD mice. After filtering for probe and segment QC, we retained 182 ROIs from *RIP-LCMV-GP* mice and 92 ROIs from NOD mice, on which 5268 genes in *RIP-LCMV-GP* mice (Supplemental Figure S8) and 19873 genes in NOD mice (Supplemental Figure S9) were quantified. In both *RIP-LCMV-GP* mice and NOD mice, all the ROIs were clustered by tissue and treatment status (Fig. 6, Fig. 7c).

Next, we investigated gene expression profiles within islets, spleen, and PLNs following TYK2 inhibition. At day 3 in *RIP-LCMV-GP* mice, we identified 102, 118, and 117 differentially expressed genes from islets, PLN, and spleen, respectively, compared to 16, 201, and 39 differentially expressed genes identified in islets, PLN, and spleen at day 7. Notably, in *RIP-LCMV-GP* mice at day 3 post-LCMV administration, there was a significant upregulation of genes associated with autophagy (*Bag3*) and anti-inflammatory genes (including *Kcp*) in islets, PLN, and spleen, suggesting a potential enhancement in cellular self-regulation and attenuation of inflammation in response to TYK2 inhibition (Fig. 6f). In contrast, expression of genes linked with tissue damage (*Reg3b*), pancreatitis (*Prss2*), and p53-mediated apoptosis (*Peg3*) were decreased in TYK2i-treated mice (Fig. 6f), suggesting activation of protective mechanisms. Moreover, an upregulation of several ribosomal genes (*Rack1*, *Rpl8*, *Rps3*, *Rps9*, *Rps16*, *Rpl13a*, and *Rps21*) in the spleen and PLN of the TYK2i-treated group was observed (Fig. 6f), suggesting that TYK2 inhibition may impact stress-mediated translational responses.

At day 7 post-LCMV inoculation, the differences in gene expression profiles persisted, indicating a lasting impact of TYK2 inhibition (Fig. 6g). In islets, PLN, and spleen, TYK2i-treated mice exhibited a notable decrease in the expression of sterile alpha motif (*Samd4b*), which is involved in the regulation of the transcriptional activity of p53, p21, and chemokine receptor 4.^80^ Importantly, there was downregulation of *Eef2*, which is linked to T cell exhaustion, in the PLN and spleen of TYK2i-treated mice. This finding was in line with our observation of increased CD4^+^PD-1^+^ and CD8^+^PD-1^+^ T cells in the flow cytometry analysis of these tissues. Interestingly, TYK2 inhibitor treatment decreased the expression of RNA binding protein (*Pabpc1*)^81^^,^^82^ in PLN and spleen (Fig. 6g), suggesting decreased ER stress and further emphasising the beneficial role of TYK2 inhibition in immune and stress regulation.

Pathway analysis of differentially expressed genes from the islets, PLN, and spleen at days 3 and 7 post-LCMV inoculation suggested that TYK2 inhibition prevented the activation of major inflammatory pathways such as EIF2 signalling, microRNA biogenesis signalling, and senescence signalling (Fig. 6h-m). There was an activation of coronavirus pathways in all tissues of the TYK2i-treated mice, potentially indicating a lower efficiency in LCMV viral clearance.

We next extended our investigation to NOD mice (Fig. 7). In this model, TYK2i treatment resulted in the downregulation of genes associated with innate immune cell proliferation (*Hemgn*, *Mcemp1*, and *En2*).^83^, ^84^, ^85^, ^86^, ^87^ In addition, there was increased expression of genes associated with the negative regulation of the Rho signalling pathway (*Arhgap4* and *Arhgap45*),^88^^,^^89^ which has been shown to modulate immune cell viability, glucose uptake, and lactate release (Fig. 7d). Similar to our findings in the *RIP-LCMV-GP* model, we observed upregulation of autophagy-related genes (*Atg12*) and genes involved in the negative regulation of inflammation (*Erdr1*) in islets and PLN of TYK2i-treated mice (Fig. 7e).

To gain insight into the functional implications of TYK2 inhibition in NOD mice, we performed an Ingenuity Pathway Analysis (Fig. 7f). Within islets, TYK2i treatment led to the downregulation of major inflammatory pathways, including neuroinflammatory signalling, pathogen-induced cytokine storm signalling, S100 family signalling, CREB signalling, and Gɑs signalling. Conversely, our analysis of PLN revealed the downregulation of pathways regulating apoptosis and DNA damage in immune cells (RXR signalling and PARP signalling). Most intriguingly, TYK2i treatment led to the upregulation of NK cell signalling and NGF and HGF signalling pathways in PLN (Fig. 7f). These findings suggest that inhibition of TYK2 modulates immune responses within lymph nodes in NOD mice.

### Spatial deconvolution analysis shows that TYK2 inhibition modulates immune cell populations in T1D models

Next, we employed spatial deconvolution analysis to determine the evolving dynamics of immune regulation in response to TYK2 inhibition in islets, PLNs, and spleen at days 3 and 7 during the evolution of LCMV-induced T1D in the *RIP-LCMV-GP* mice (Fig. 6n–s and Supplemental Figure S8e–j). On day 3 post-LCMV injection, we observed a significant reduction in gamma delta (ɣδ) T cells (P = 0.0004, Mann–Whitney test) and a decline in stem and progenitor cells (P = 0.0021, Mann–Whitney test) (Supplemental Figure S8e). Simultaneously, a shift in immune cell populations was seen in the spleen and PLNs, where there was a substantial decrease in B cell populations (P = 0.0122) and an increase in DCs (P = 0.0035) in PLN and spleen (B cell, P = 0.0005; DCs, P = 0.0003) of TYK2i-treated mice. However, there was a significant decrease in activated T cells in PLN (P < 0.0001) at day 3 in TYK2i-treated mice. Furthermore, TYK2i treatment increased ɑβ T cells in spleen (P = 0.0032) and induced a significant decrease in ɣδ T cells (P = 0.0097) in the spleen at day 3, suggesting that TYK2 inhibition results in complex immune cell modulation in multiple target tissues (Supplemental Figure S8i).

On day 7 post-LCMV injection, we observed a substantial increase in ɑβ T cells (P = 0.0001), innate lymphoid cells (ILCs) (P = 0.0136), monocytes (P = 0.0142), myeloid cells (P = 0.0229), and stromal cells (P = 0.0253) within islets of TYK2i-treated mice. In the spleen and PLN, there were profound alterations in immune cell profiles, with an increase in DC, ILC, myeloid cell, stem and progenitor cell, and stromal cell populations. Consistent with the day 3 effects, there was a significant decrease in ɣδ T cell (P = 0.0003) populations in the spleen of TKY2i-treated mice on day 7 (Supplemental Figure S8j).

In agreement with the above observations in the *RIP-LCMV-GP* model, data from NOD mice corroborated further the effects of TYK2i treatment on the tissue immune cell repertoire (Fig. 7i and j and Supplemental Figure S9). A significant decrease in activated T cells was noted in islets and PLN of TYK2i-treated mice, accompanied by an increase in DCs (islets, P = 0.0215; PLN, P < 0.0001), ILCs (islet, P = 0.0002; PLN, P < 0.0001), myeloid cells (islet and PLN, P < 0.0001), stem and progenitor cells (islet and PLN, P < 0.0001), stromal cells (islet and PLN, P < 0.0001), ɑβ T cells (islet, P < 0.0001; PLN, P = 0.0016), and ɣδT cells (PLN, P < 0.0001, Mann–Whitney test) (Fig. 7i and j). This consistency in immune cell population dynamics across two mouse models of T1D underscores the robustness of the effects induced by TYK2 inhibition across the pancreas, PLN, and spleen.

### Spatial proteomics show that TYK2 inhibition reshapes immune dynamics in T1D models

**Fig. 8:**
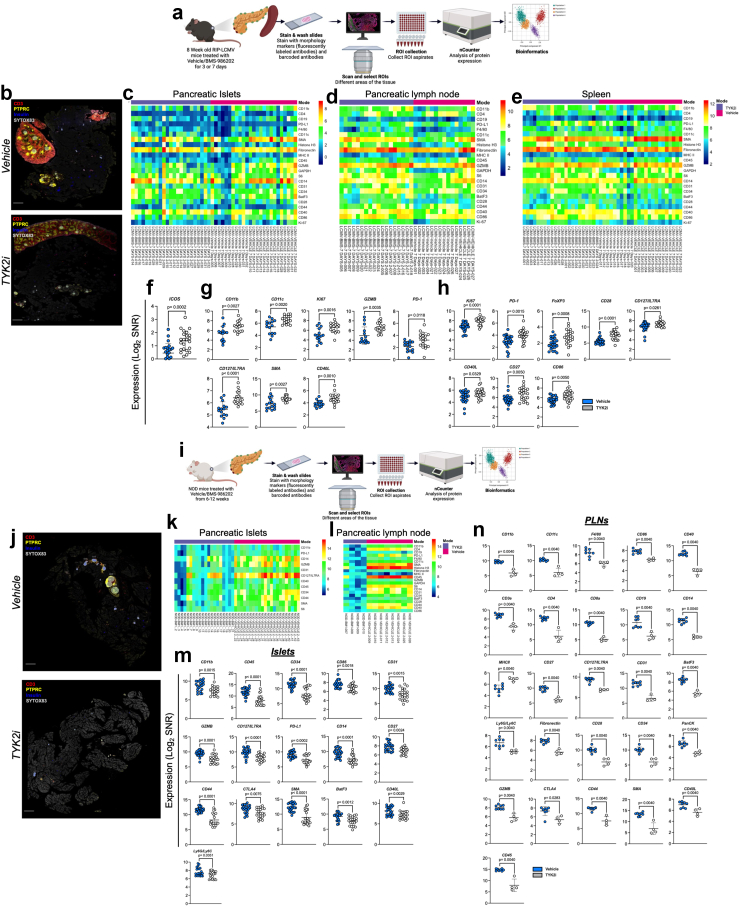
**Spatial proteomics uncover immune cell dynamics in TYK2i-treated *RIP-LCMV-GP* and NOD mouse models**. Tissues were harvested from vehicle- and TYK2i-treated *RIP-LCMV-GP* mice on day 7 post-inoculation. (**a**) Schematic representation of GeoMx-DSP proteomics experimental workflow from RIP-LCMV-GP mice. (**b**) Representative images of islets, PLN, and spleen labelled for CD3 (red), PTPRC (yellow), insulin (blue), and Sytox83 (grey). (**c–e**) Heatmap showing the overall expression of immune cell typing and immune cell activation makers from selected ROIs in (**c**) islets, (**d**) PLN, and (**e**) spleen. (**f–h**) Markers that show a significant difference between vehicle- and TYK2i-treated mice in (**f**) islets, (**g**) PLN, and (**h**) spleen. (**i**) Schematic representation of GeoMx-DSP proteomics experimental workflow for tissues from NOD mice. (**j**) Representative images of islets and PLN labelled for CD3 (red), PTPRC (yellow), insulin (blue), and Sytox83 (grey). (**k–l**) Heatmap showing the overall expression of immune cell typing and immune cell activation markers from selected ROIs in (**k**) islets and (**l**) PLN. (**m, n**) Markers that showed significant differences between vehicle- and TYK2i-treated mice from (**m**) islets and (**n**) PLN; n = 4 mice/group for RIP-LCMV-GP mice and n = 3–4 for NOD mice per group. Data were normalised to the geometric mean of the IgG negative control, and the significantly differently expressed proteins were presented as Log_2_ of signal-to-noise ratio (SNR). Differences between groups were determined by Mann–Whitney U tests, and the data are presented as mean ± SD.

To substantiate our findings, we performed GeoMx spatial proteomics profiling on islets, PLNs, and spleen collected from *RIP-LCMV-GP* mice at 3 and 7 days post-LCMV administration (Fig. 8a–h and Supplemental Figure S10) and on islets and PLN from NOD mice at 12 weeks of age (Fig. 8i-n). Tissue sections from 3 to 4 mice per group were stained with CD3, B220, insulin, and Sytox83 (nucleus) to identify ROIs. At day 3 post-LCMV infection, no discernible differences were observed in the proportion of immune cells or markers of immune cell activation between the vehicle- and TYK2i-treated *RIP-LCMV-GP* mice (Supplemental Figure S10). However, by day 7 post-LCMV infection, in TYK2i-treated mice, there was a significant increase in the proportion of cells positive for CD11b (P = 0.0027) and CD11c (P = 0.002) in PLN, and there was also an increase in the number of cells expressing Ki67 (PLN, P = 0.0016; spleen, P < 0.0001), CD127/IL7RA (PLN, P < 0.0001; spleen, P = 0.0261), and CD40L (PLN, P = 0.0010; spleen, P = 0.0329) in PLN and spleen compared to vehicle-treated mice. Notably, a heightened presence of FOXP3-positive cells (P = 0.0008) was noted in the spleen of TYK2i-treated mice. We also observed an upregulation of PD-1 expression (PLN, P = 0.0118; spleen, P = 0.0015) in the PLN and spleen of TYK2i-treated mice (Fig. 8f–h). These findings are consistent with our flow cytometry and spatial deconvolution data and collectively suggest that TYK2 inhibition in *RIP-LCMV-GP* mice kerbs T cell activation while leaving innate immune cell profiles largely unaffected.

In NOD mice treated with TYK2i there was a substantial reduction in markers of immune cell activation and immune cell populations in islets and PLN (Fig. 8i-n), with a significant decrease in the expression of innate and adaptive immune cell markers (CD11b (islets, P = 0.0015; PLNs, P = 0.0040), CD11c (PLNs, P = 0.0040), F4/80 (PLNs, P = 0.0040), CD86 (islets, P = 0.0018; PLNs, P = 0.0040), CD40 (PLNs, P = 0.0040), CD3e (PLNs, P = 0.0040), CD4 (PLNs, P = 0.0040), CD8a (PLNs, P = 0.0040), CD19 (PLNs, P = 0.0040), CD14 (PLNs, P = 0.0040), CD27 (islets, P = 0.0024; PLNs, P = 0.0040), CD127/IL7RA (PLNs, P = 0.0040), CD31 (islets, P = 0.0015; PLNs, P = 0.0040). In addition, we also observed a significant decrease in the expression of markers of T cell activation (Ly6G/Ly6C (islets, P = 0.0351; PLNs, P = 0.0040), Fibronectin (PLNs, P = 0.0040), CD28 (PLNs, P = 0.0040), CD34 (islets, P < 0.0001; PLNs, P = 0.0040), PanCK (PLNs, P = 0.0040), GZMB (islets, P < 0.0001; PLNs, P = 0.0040), CTLA4 (islets, P < 0.0075; PLNs, P = 0.0283), CD44 (islets, P < 0.0001; PLNs, P = 0.0040), SMA (islets, P < 0.0001; PLNs, P = 0.0040), CD40L (islets, P = 0.0029; PLNs, P = 0.0040), and CD45 (PLNs, P = 0.0040)) in TYK2i treated NOD mice. Despite the decreased expression of activation markers, an increase in MHCII expression was observed in PLN of NOD mice receiving TYK2i treatment (Fig. 8n). Statistical differences were assessed using Mann–Whitney test.

## Discussion

The first small molecule TYK2 inhibitor was approved by the FDA in 2022 for the treatment of psoriasis, with additional agents under investigation for use in other autoimmune conditions.^90^, ^91^, ^92^ TYK2 mediates signalling through several cytokines that have been linked with T1D pathogenesis, including IFNα, IL-12, and IL-23. Along these lines, missense mutations in TYK2 are associated with protection against T1D development, making it an appealing pharmacological target for disease intervention.^4^^,^^7^ In the present study, we demonstrate that the highly specific TYK2 pseudokinase inhibitors, BMS-986165 and BMS-986202, modulate three critical nodes in T1D pathophysiology including: 1) immune cell activation and target tissue infiltration; 2) β cell inflammation and survival; and 3) direct interaction of β cells with antigen-specific CD8+ T cells. Taken together, these data provide robust rationale for testing the effectiveness of TYK2 inhibitors in clinical trials to prevent and/or delaying the development of T1D.

We utilised three *in vitro* human experimental models (EndoC-βH1 cells, dispersed adult human islets, and pancreatic islet-like cells differentiated from human iPSCs) to study the direct effects of TYK inhibition on the β cell. We found that BMS-986165 and BMS-986202 reduced IFNα-mediated upregulation of *CHOP*, *HLA class I, MX1*, and *CXCL10* gene expression. Similarly, TYK2 inhibition reduced β cell chemokine production, ER stress, and HLA class I upregulation in response to a cocktail of cytokines that included IFNɑ+TNFɑ and IFNɑ+IL-1β. These data are consistent with a previous publication from our group using two different TYK2is^34^^,^^70^ and findings from others using BMS-986165.^93^ Importantly, we show these effects in the β cell are sufficient to reduce T cell activation in a co-culture system.

To test the *in vivo* efficacy of TYK2i, we utilised two well-established mouse models of T1D. We found that TYK2 inhibition prevents the onset of hyperglycemia in both RIP-*LCMV-GP* and NOD mice. We leveraged the predictable and rapid onset of T1D with viral induction in the *RIP-LCMV-GP* model to define the temporal effects of TYK2 inhibition. At early time points (i.e., 3 days after viral induction), flow cytometry revealed a decrease in mature NK cells in the blood of TYK2i-treated *RIP-LCMV-GP* mice, with a reciprocal increase in a subset of immature NK cells. This particular finding was notable given previous studies showing changes in NK cell phenotypes in established T1D^94^ and the recent identification of an NK-enriched signature associated with both islet autoimmunity and T1D onset in the TEDDY and DIPP cohorts, which follow newborns with high genetic risk of T1D.^95^^,^^96^ Intriguingly, a recent analysis of pancreatic draining lymph nodes and mesenteric lymph nodes from human organ donors showed an increase in cytotoxic CD56_dim_ CD16+ NK cell populations in pancreatic lymph nodes from donors with T1D—*see preprint*.^97^ Finally, we observed an increase in the percentage of CD11b+ DCs in the spleen of TYK2i-treated *RIP-LCMV-GP* mice, followed by a reduction of these cells in the PLN at day 7. Intriguingly, spatial transcriptomics indicated an increase in DC populations that expressed CD207 in the islets and PLN of TYK2i-treated mice. While the exact phenotypes and roles of DCs in T1D have been controversial, the expansion of specific DC populations, including those that express CD207, has been linked with pro-tolerogenic phenotypes.^98^^,^^99^

At later timepoints, immune profiling of TYK2i-treated *RIP-LCMV-GP* mice revealed changes that were largely restricted to adaptive immune cell subsets. In this model, flow cytometry revealed increased percentages of PD1^+^ CD4^+^ and CD8^+^ T cells in the blood, spleen, and PLN of TYK2i-treated mice, while spatial transcriptomics showed decreased ɣ/δT-cells in the islets, spleen, and PLN. Consistent with changes in effector cell subsets, transcriptomics in TYK2i-treated NOD mice revealed decreased expression of markers of immune cell activation (CD86, GZMB, CD127/IL7RA, CD44, CTLA4, SMA, BatF3, CD40L, and Ly6G/Ly6C) in both islets and PLN, coupled with reduced insulitis. The effects of TYK2i on PD1-expressing T cell subsets were striking, as chronic activation of PD1 is linked with T cell exhaustion characterised by reduced T cell activation,^100^ diminished cytokine production, and reduced cytotoxic activity.^101^ Importantly, the most effective immune modulator for T1D prevention to date, Teplizumab, is also associated with an exhausted T cell phenotype,^102^ and T cell exhaustion has also been linked with response to anti-thymocyte globulin (ATG) treatment in Stage 3 T1D.^103^^,^^104^ Intriguingly, we observed an increase in FoxP3^+^PD1^+^ Tregs in the spleen and PLN in TYK2i-treated *RIP-LCMV-GP* mice. Alterations in Treg function are a well-described component of T1D pathophysiology^105^; however, relatively few studies have characterised the frequency or function of PD1^+^ expressing Tregs in models of diabetes. NOD mice with inducible deletion of PD1^+^ were protected against diabetes,^106^ suggesting that PD1 may restrain Treg function in models of autoimmunity. In contrast, in cancer models, PD1-expressing Tregs possess potent immunosuppressive effects and are associated with resistance to checkpoint inhibitors. In human cohort studies, the ratio between PD1^+^ effector and regulatory subsets has been proposed as a biomarker of cancer drug response,^107^ indicating there is complexity in interactions across different immune cell subsets that may not be appreciated when PD1^+^ is deleted in a single immune cell type.

At the level of the pancreas, *in vivo* TYK2i treatment modulated expression patterns of interferon-stimulated (ISG) genes, with reduced *CD274, MX1, CXCL10*, and *STAT1* in *RIP-LCMV-GP* mice and reduced *Mx1, CXCL10*, and *STAT1* in NOD mice. TYK2i treatment was associated with changes in the subcellular localisation of key ISGs. Most notably, cytoplasmic CD274 mRNA was increased in TYK2i-treated *RIP-LCMV-GP* mice at day 14 and in NOD mice, suggesting that changes in subcellular localisation of mRNAs may be influenced by reducing inflammation.^77^, ^78^, ^79^^,^^108^ Of note, changes in mRNA localisation have been linked with changes in mRNA translation in several disease models.^78^^,^^109^ In addition, mRNA localisation is influenced by specific RNA binding proteins.^78^^,^^110^ We have shown previously that pro-inflammatory cytokines can impact the expression of RNA binding proteins in β cells.^58^ Interestingly, PDL-1 protein was reduced in islets from TYK2i-treated *RIP-LCMV-GP* mice at day 3 and 7, but levels were not different between vehicle- and TYK2i-treated mice at day 14. Similar expression patterns of PDL-1 were observed in NOD mice.

Spatial transcriptomics allowed us to compare tissue-specific signatures across the islets, PLNs, and spleen and probe novel pathways through which TYK2is may impact the islet. The number of differentially expressed genes was similar across these tissues, highlighting the potential of this class of drugs to modulate signalling in both the immune and endocrine compartments. Pathway analysis highlighted downregulation of pathways related to EIF2A signalling, S100A signalling, and senescence in TKY2i-treated *RIP-LCMV-GP* mice, while inflammatory pathways, including CREB and Gαs signalling, were downregulated in NOD mice treated with TYK2i.

In a recent publication,^36^ Mine et al. studied the phenotype of NOD mice with total body TYK2 deletion and found that these mice were protected against T1D development and exhibited decreased activation of autoreactive CD8^+^ T-BET^+^ CTLs due to reduced IL-12 signalling in CD8^+^ T cells. Additionally, in a limited pharmacological prevention study, the authors found that NOD mice treated with TYK2i BMS-986165 from 6 to 10 weeks of age were partially protected from diabetes development. Importantly, the major findings of this study are in agreement with our observations, underscoring the reproducibility and clinical potential of modulating this pathway. However, our study also characterises how TYK2is impact β cell stress pathways and how they modulate innate and adaptative immune responses at multiple levels, from the transcriptome to the proteome, with detailed longitudinal phenotyping, thus adding substantially to our understanding of the role of this pathway in the development of T1D. Furthermore, our validation in three human β cell models and two mouse models support the translation of these agents for clinical testing in polygenic and heterogenous populations.

We acknowledge that several key questions remain as to the ideal timing and chronicity of intervention with TYK2 inhibitors. For example, should testing of TYK2is be prioritised in at-risk individuals (i.e. Stage 1 or 2) or after Stage 3/clinical disease onset? The fact that an IFN signature is present in β cells in both the pre-diabetic period and after disease onset^10^^,^^96^ suggests that treatment at earlier timepoints could be more efficacious to preserve β cell mass and function. However, the recent report that a JAK inhibitor was able to preserve C-peptide levels in Stage 3 T1D^111^ provides support for initial testing of TYK2i after clinical disease onset. Second, the duration of therapy is an important consideration for cytokine inhibitors. Data from our preclinical studies suggest that there is modulation of the adaptive immune cell repertoire, but whether this is a persistent effect remains to be tested. In our study, the *RIP-LCMV-GP* mice were followed for 14 days post LCMV inoculation, and the NOD mice were followed until 25 weeks of age. These timepoints were appropriate for our study; however, it will be informative to determine the impact of TYK2 inhibition during longer periods of follow-up, as the application of JAK and TYK2 inhibitors in other autoimmune conditions suggests chronic dosing would likely be necessary.^112^^,^^113^ An additional limitation of our study is that spatial transcriptomics and proteomics studies had smaller samples sizes compared to other endpoint studies. Notwithstanding these unresolved questions, our results underscore the diverse and beneficial effects of TYK2 inhibition at the level of the β cell and the immune system in pre-clinical models of T1D, strongly supporting the rationale for testing these agents as a novel therapy to preserve β cells in T1D.

## Contributors

FS, CEM, and DLE conceived and designed the study. FS, OB, CCL, JR, AC, SAW, ST, SD, ACdB, MIA, KO, AZ, EMV and DS performed experiments. PK, NSC, GC, and JL performed computational and statistical analyses FS, PK, NC, and JK. FS, CEM, and DLE interpreted the data. FS and CEM wrote the manuscript. LM and PM provided human islets for research. All authors provided critical revisions and edits to the manuscript. All authors read and approved of the final manuscript. CEM is the guarantor of this work. Both FS and CEM have verified the underlying data of this manuscript.

## Data sharing statement

Data presented in this manuscript are available from the corresponding author CEM upon request. Genomic data generated for this manuscript has been deposited into the Genome Expression Omnibus Repository under accession number GSE262211. Reviewers can access the data using the following reviewer token: ubmhgyegtzsjnul. The proteomics data generated for this manuscript will be deposited at the time of publication.

## Declaration of generative AI and AI-assisted technologies in the writing process

During the preparation of this work, the author used ChatGPT in order to organise the flow of the manuscript text. After using this tool/service, the author reviewed and edited the content as needed and takes full responsibility for the content of the publication.

## Declaration of interests

CEM has received grants from Lilly Pharmaceuticals and Astellas Pharmaceuticals (not related to this manuscript). CEM has served on advisory boards related to T1D research clinical trial initiatives: Isla Technologies, Neurodon, and DiogenX. CEM has patent (16/291,668) Extracellular Vesicle Ribonucleic Acid (RNA) Cargo as a Biomarker of Hyperglycaemia and Type 1 Diabetes and CEM and FS have a provisional patent (63/285,765) Biomarker for Type 1 Diabetes (PDIA1 as a biomarker of β cell stress). DLE serves on the advisory board of InSphero, related to the preparation of islet microtissues. EMV received grant from Fonds de la Recherche. JSK has received grants from NIDDK—U24DK104162, R21DK127285 and grants from (Breakthrough T1D–5-SRA-2018-557-Q-R) and Helmsley Charitable Trust–2018 PG-T1D053 and G-2108-04793). JSK received support to attend NIDDK study section, as well as to attend the NIDDK sponsored AI meeting October 2024. SAW is employed by Eli Lilly and owns stocks in the company and was supported by the company to attend a conference. SAW is a board member of Breakthrough T1D. FS received honoria and a travel award to present current work at ADA conference. These activities have not dealt directly with topics covered in this manuscript.

## Appendix A Supplementary data

Supplemental Figures and Legends

Supplemental Western Blots

Reagent validation

Star Methods

TYK2i_Raw Data
