# Supplementary material for: Pharmacological inhibition of tyrosine protein-kinase 2 reduces islet inflammation and delays type 1 diabetes onset in mice
Source: eBioMedicine. 2025 May 6;117:105734. doi: 10.1016/j.ebiom.2025.105734 (PMC12173048; doi:10.1016/j.ebiom.2025.105734)
Supplement: Supplemental Figures and Legends [file mmc1.docx]

**Supplemental Figure Legends:**


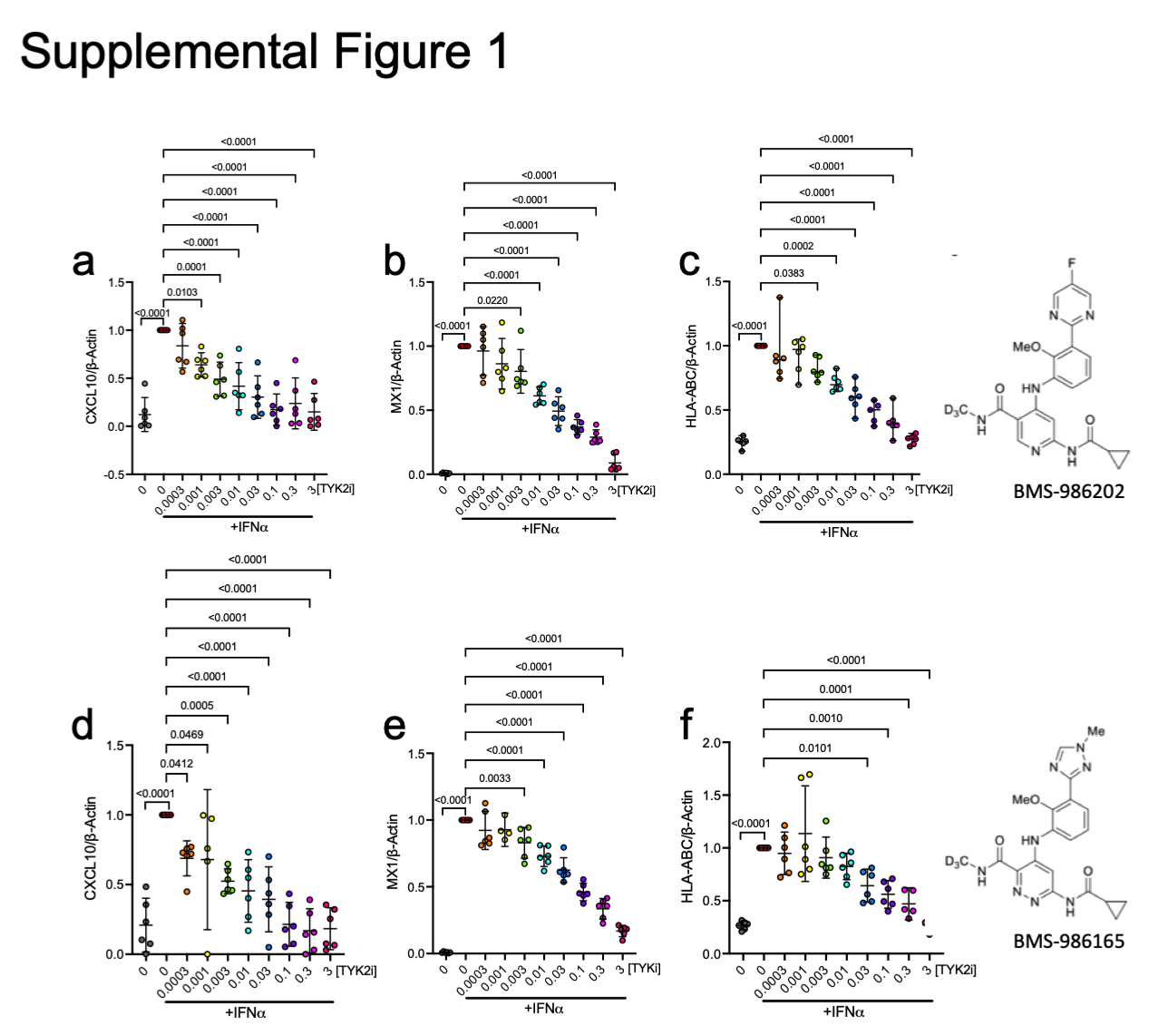


**Supplemental Figure 1. TYK2 inhibitors BMS-986202 and BMS-986165 alleviate IFNα-induced gene expression in EndoC-βH1 cells.**

(**a-f**) Cells were pre-treated for 2 h with the indicated concentrations (expressed in mM) of (**a-c**) BMS-986165 or (**d-f**) BMS-986202 and exposed to IFNα (2000 U/mL) in the continued presence of the inhibitors for 24 h. mRNA expression of (**a,d**) *CXCL10*, (**b,e**) *MX1*, and (**c,f**) *HLA-ABC* was analyzed by qPCR. Data were normalized to β-actin expression level and expressed as fold changed compared to cells exposed to IFNα alone. Data are presented as mean with 95% CI for 6 independent experiments with individual data indicated. A one-way ANOVA followed by Dunnett’s test multiple comparisons was used for statistical analysis.


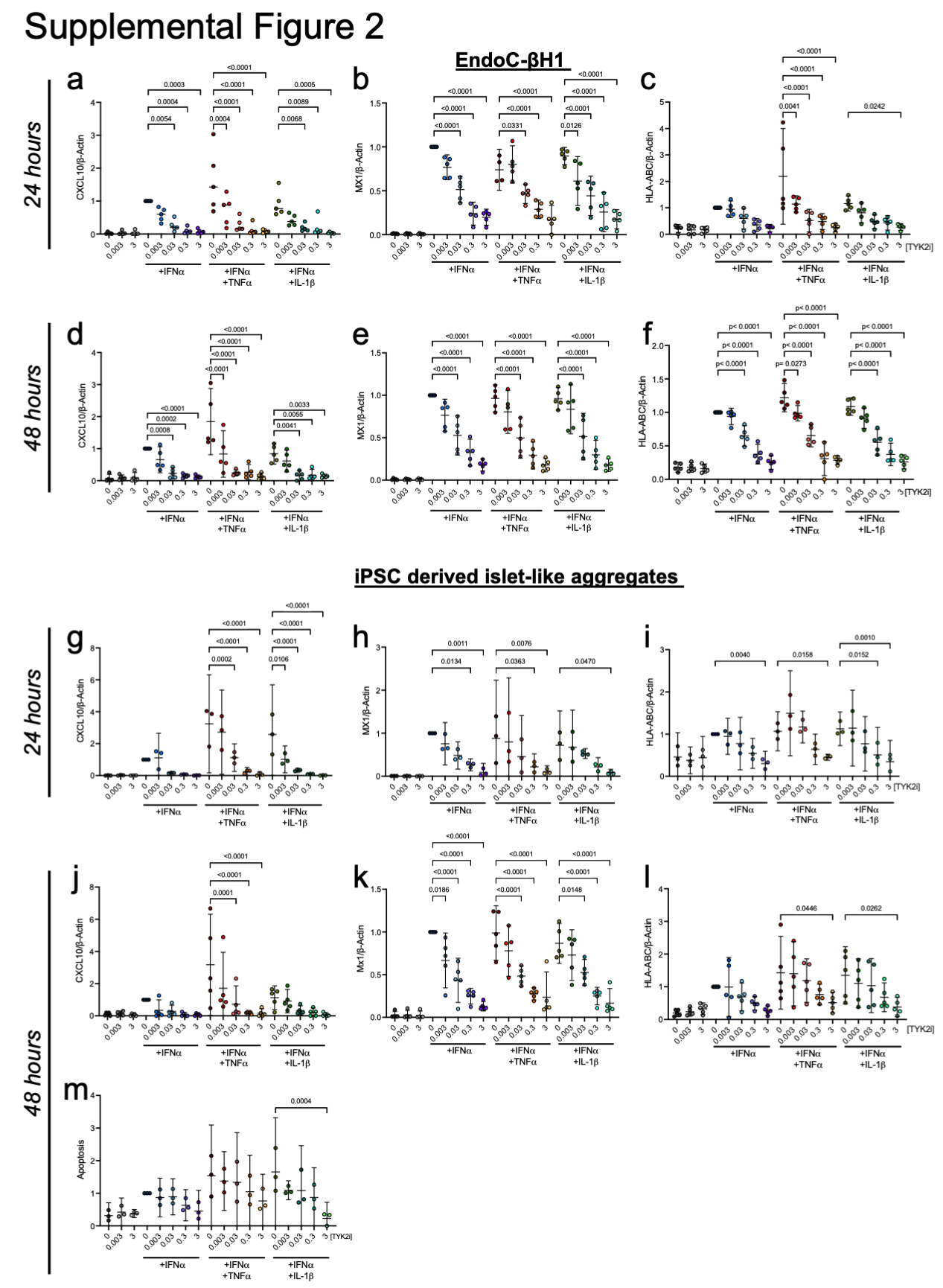


**Supplemental Figure 2. TYK2 inhibition suppresses cytokine-induced gene expression in human β cells and iPSC-derived β-like cells.**

(**a-f**) EndoC-βH1 cells were pre-treated for 2 h with the indicated concentrations (expressed in mM) of BMS-986202 prior to exposure to IFNα (2000 U/mL), IFNα (2000 U/mL) + TNFα (1000 U/ml), or IFNα (2000 U/mL) + IL-1β (50 U/mL) in the continued presence of the TYK2i for **(a-c)** 24 h or **(d-f)** 48 h. mRNA expression of (**a,d**) *CXCL10*, (**b,e**) *MX1*, and (**c,f**) *HLA-ABC* was analyzed by RT-qPCR. (**g-i**) Control iPSCs (HEL115.6) were differentiated into islet-like cells using a previously described protocol (41) and pre-treated for 2 h with the indicated concentrations (expressed in mM) of BMS-986202 prior to exposure to IFNα (2000 U/mL), IFNα (2000 U/mL) + TNFα (1000 U/ml), or IFNα (2000 U/mL) + IL-1β (50 U/mL) in the continued presence of the TYK2i for (**g-i**) 24 h or (**j-m**) 48 h. mRNA expression of (**g, j**) *CXCL10*, (**h, k**) *MX1*, and (**i, l**) *HLA-ABC* was analyzed by qPCR. Islet-like aggregates were dissociated and seeded in 8-well chamber slides. After 48 h, cells were pre-treated for 2 h with the indicated concentrations (expressed in mM) of BMS986202 and exposed to IFNα (2000 U/mL), IFNα (2000 U/mL) + TNFα (1000 U/mL), or IFNα (2000 U/mL) + IL-1β (50 U/mL) in the continued presence of inhibitor for 48 h. (**m**) Apoptotic cells were identified by Hoechst 3342 and propidium iodide staining. For qPCR analysis, data were normalized for β-actin expression and expressed as fold change from cells exposed to IFNα alone. Data are presented as means with 95% CI, n= 5 for EndoC-βH1 cells. For iPSC cells, n=3 for 24 h and n=6 for 48 h timepoints. Independent experiments with individual data are indicated. For apoptosis studies in iPSC derived islet-like aggregates, data are expressed as fold change compared to cells exposed to IFNα and are presented as mean ± SEM for 3 independent experiments with individual data indicated. A one-way ANOVA followed by Sidak’s multiple comparisons test was used for statistical analysis.


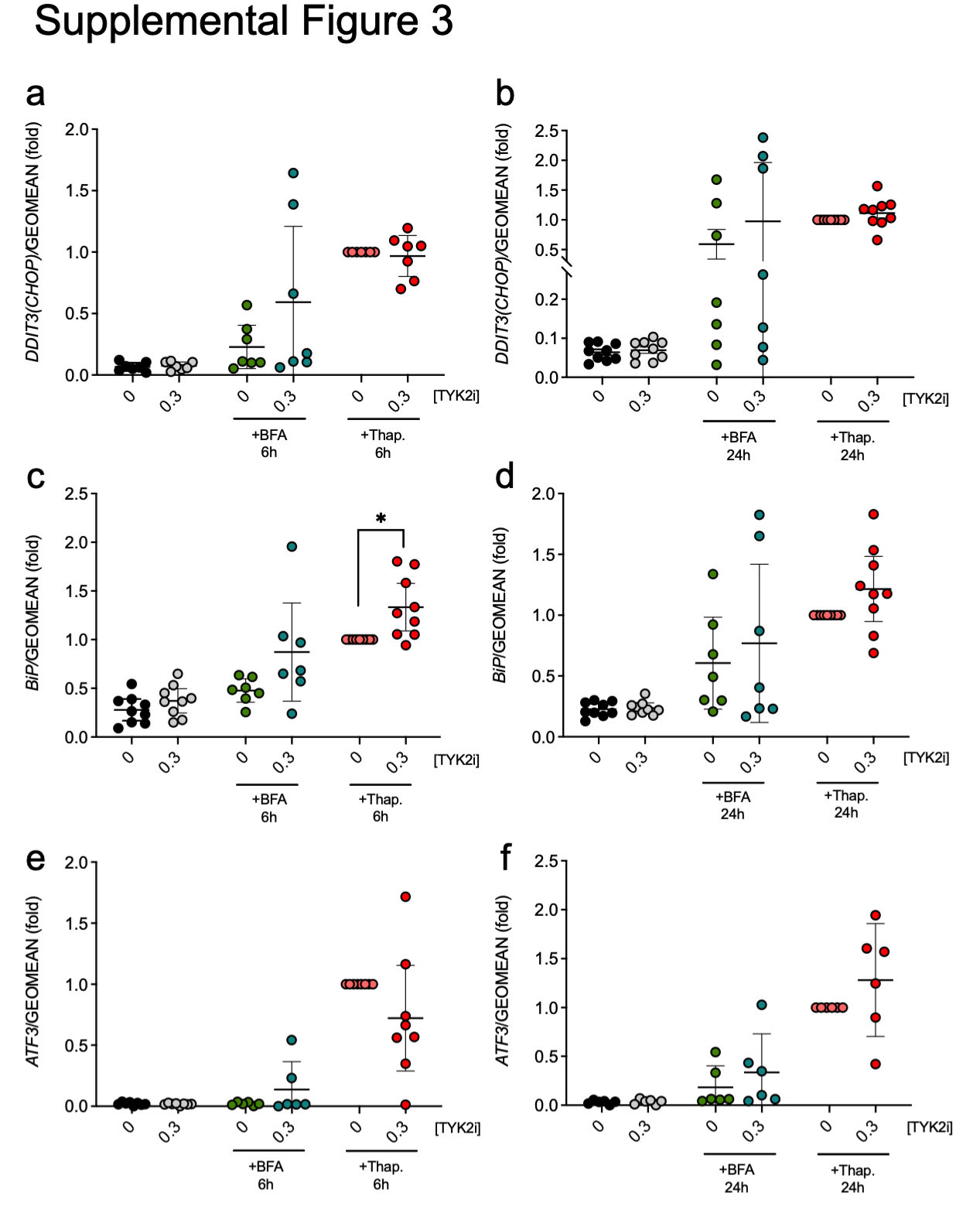


**Supplemental Figure 3. TYK2 inhibition does not rescue endoplasmic reticulum stress induced by BFA or Thapsigargin.** EndoC-βH1 cells were pre-treated for 2 h with the indicated concentration (expressed in mM) of TYK2i BMS-986165. Treatment was continued with BMS-986165 in the absence or presence of brefeldin A (BFA, 0.02 μg/mL) or thapsigargin (Thap, 1μM) for 6 h or 24 h, and mRNA levels of (**a,b**) *DDIT3 (CHOP),* (**c,d**) *ATF3* and (**e,f**) *BiP* were analyzed by RT-qPCR, normalized to the geometric mean of *ACTB* and *VAPA,* and expressed relative to thapsigargin-treated cells. Results are means with 95% CI. Each point represents an independent experiment. One-way ANOVA with Bonferroni correction was used to determine statistical difference.


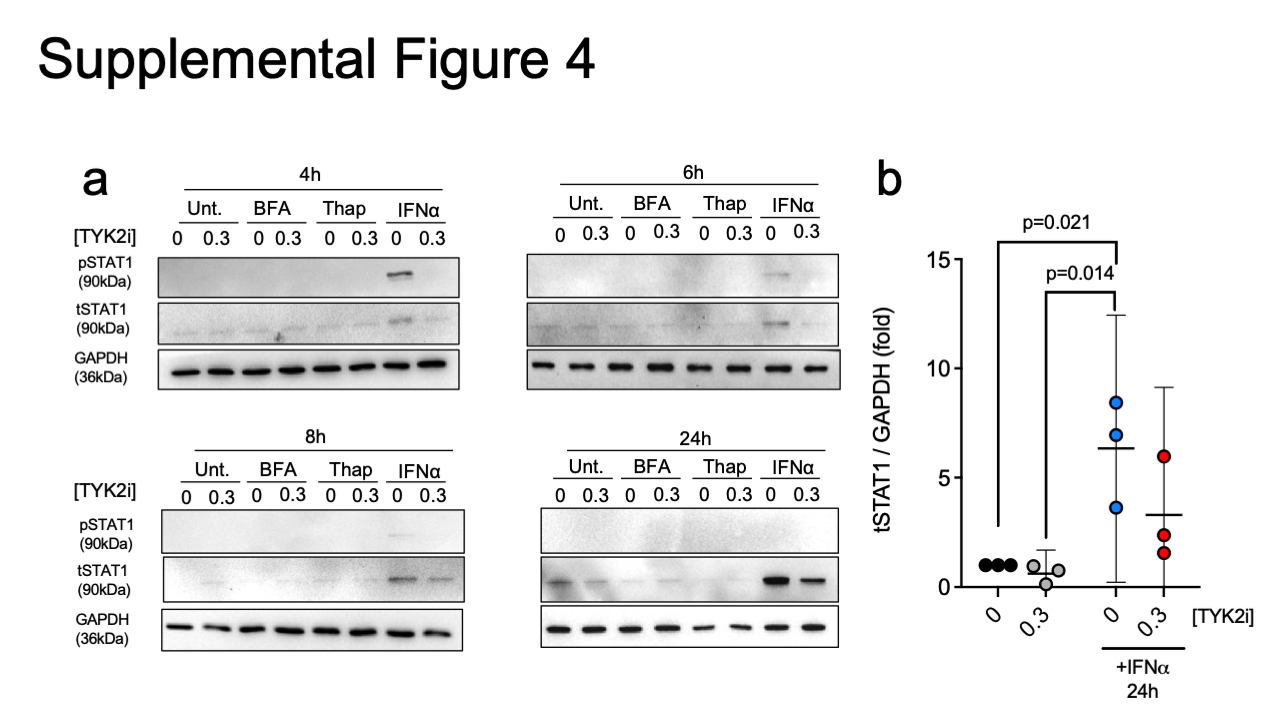


**Supplemental Figure 4. Chemical ER stressors do not induce pSTAT1 or tSTAT1; BMS-986165 decreases IFNα-induced upregulation of pSTAT1 and tSTAT1.** EndoC-βH1 cells were pre-treated for 2 h with the indicated concentration (expressed in mM) of TYK2i BMS-986165. Treatment was continued with BMS-986165 in the absence or presence of brefeldin A (BFA, 0.02 μg/mL), thapsigargin (Thap, 1μM), or IFNα (2000 U/mL) for 4, 6, 8, or 24 h. (**a**) Western blotting of protein lysates was performed to detect pSTAT1 and tSTAT1 after 24h of treatment with IFN⍺ or TYK2i. (**b**) Data were normalized to the levels of GAPDH and expressed relative to non-treated cells. Results are means with 95% CI. Each point represents an independent experiment. One-way ANOVA with Tukey multiple comparison was used to determine statistical difference.


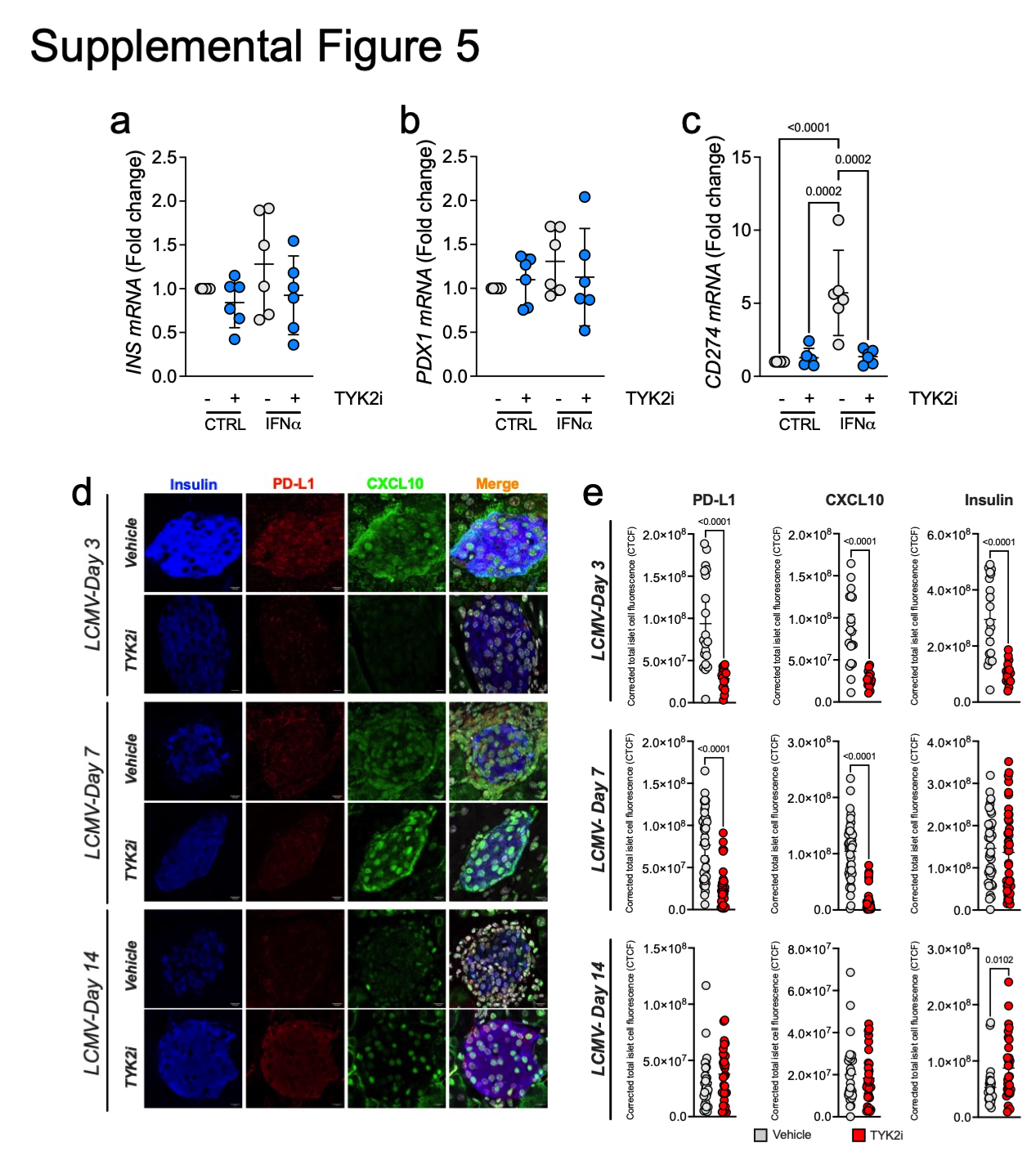


**Supplemental Figure 5. TYK2 inhibition does not affect mRNA expression of β cell-specific genes but inhibits *CXCL10* and *CD274* expression.**

(**a-c**) Human islets were pretreated with DMSO or BMS-986165 (0.3 µM) for 2 h and then co-incubated with or without IFNα (2000 IU/mL) for 24 h. qPCR analysis of (**a**) *INS*, (**b**) *PDX1*, and (**c**) *CD274*. Results are presented as mean with 95% CI for 5 independent experiments with individual data indicated. (**d-e**) Pancreatic tissue was harvested from vehicle- and TYK2i-treated *RIP-LCMV-*GP mice on days 3, 7, and 14 post-inoculations. (**d**) Representative immunofluorescence images of PD-L1 (red) and CXCL10 (green) in pancreatic islets at 3-, 7-, and 14-days post-inoculation. (**e**) Quantification of immunofluorescence signals presented as corrected cellular fluorescence intensities. Grey and red circles indicate vehicle- and TYK2i-treated groups, respectively. Data are expressed as mean with 95% CI with individual data presented. For qPCR assay, a one-way with multiple comparison was used; for immunofluorescence studies, n=5-7 mice per condition and 4-7 islets per section and statistical significance was determined by a Mann-Whitney U test.


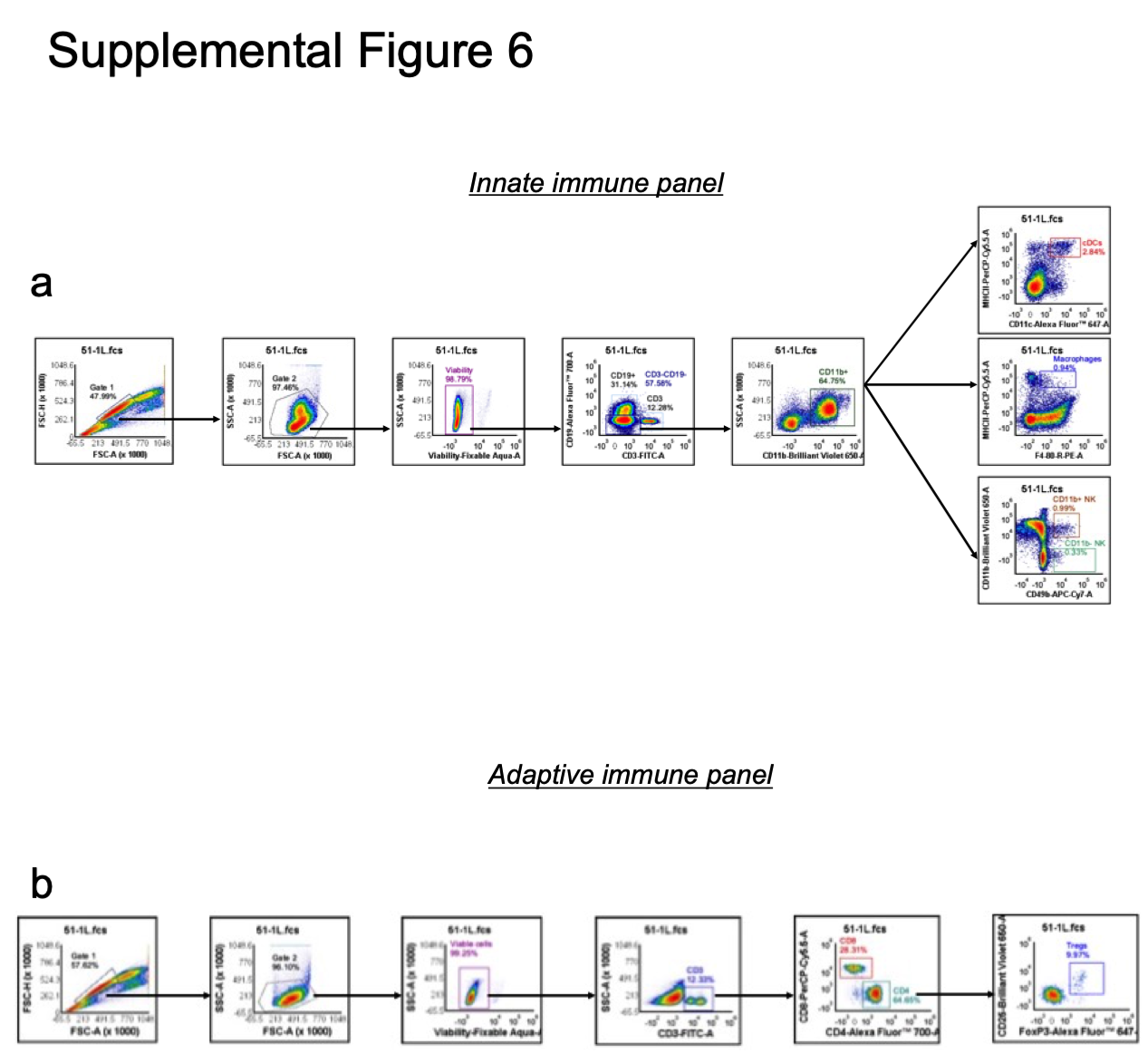


**Supplemental Figure 6. Gating strategy for flow cytometry analysis from *RIP-LCMV-GP* mice.**

Representative images of scatter plots showing immune cell markers used to determine the abundance of (**a**) innate and (**b**) adaptive immune cells in the blood, PLN, and spleen of *RIP-LCMV-GP* mice.


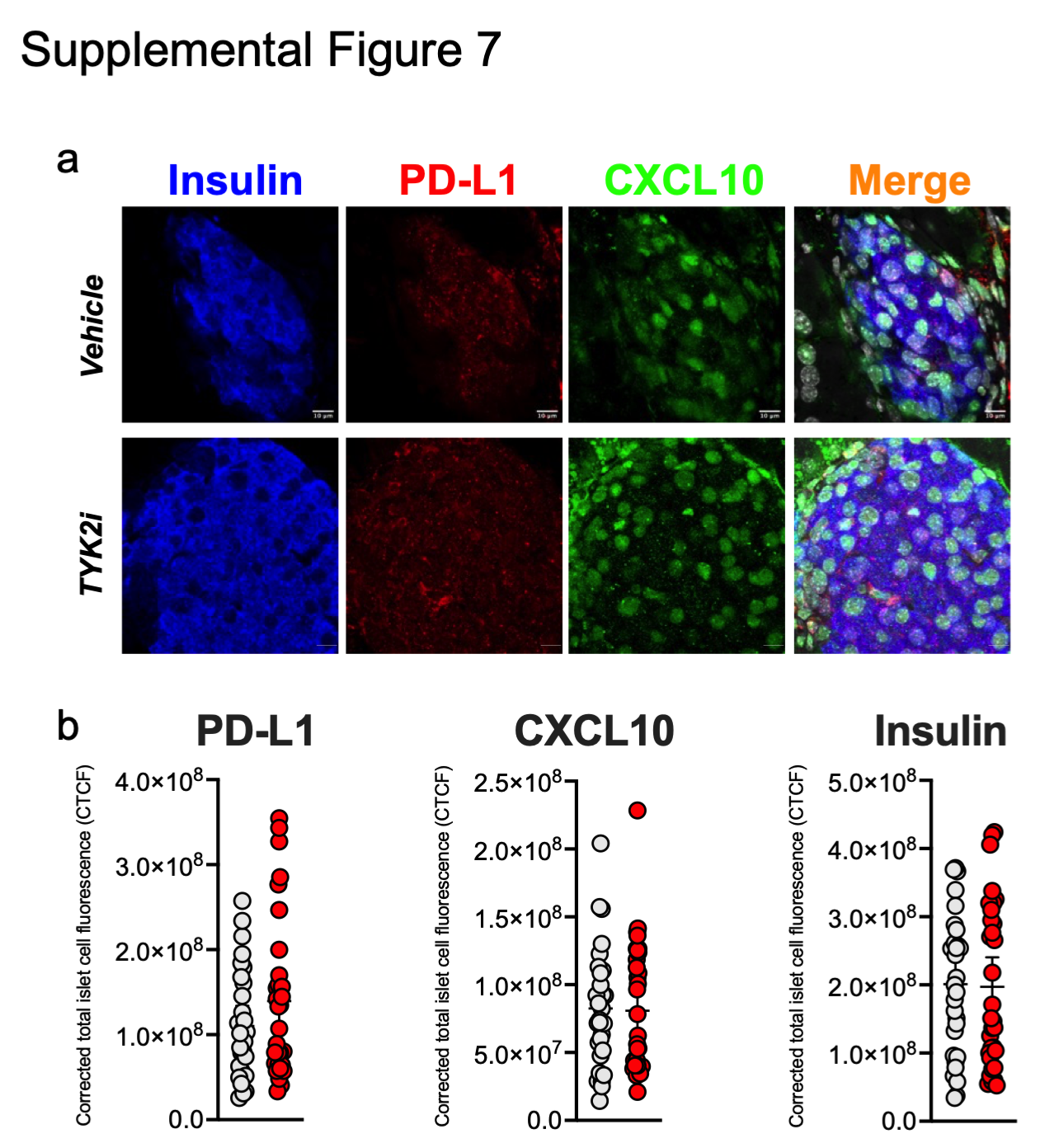


**Supplemental Figure 7. TYK2 inhibition does not affect IFNα-induced PD-L1 and CXCL10 protein expression in islets of prediabetic NOD mice.**

Pancreas tissue was harvested from vehicle- or TYK2i-treated NOD mice at 13 weeks of age. (**a-b**) Immunofluorescent labeling for the detection of insulin, PD-L1, and CXCL10 in pancreas tissue sections. (**a**) Representative confocal images and (**b**) corrected total cellular fluorescence intensities from islets of vehicle- and TYK2i-treated NOD mice. Grey and red bars indicate measures in tissues from vehicle- or TYK2i-treated mice, respectively. Data are expressed as mean with 95% CI with individual data presented, n=4-6 mice per condition: statistical significance was determined by Mann-Whitney U test.


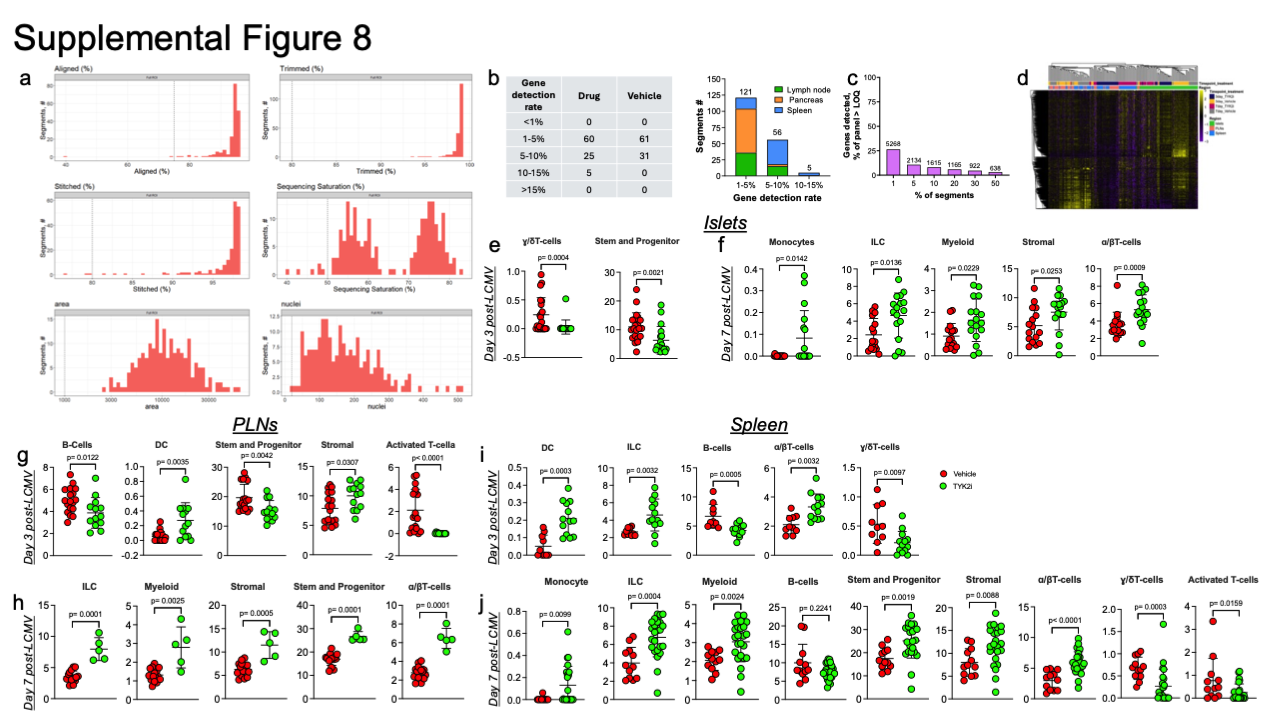


**Supplemental Figure 8. Quality control and normalization strategies used in spatial whole transcriptome analysis of *RIP-LCMV-GP* mice.**

(**a**) Percentage of aligned sequencing read counts at every step of sequencing and sequencing saturation. (**b**) Gene detection rate across different cutoff ratios and (**c**) number of genes detected across different filtering criteria. (**d**) Heatmap showing gene clustering using genes with a high coefficient of variation. (**e-j**) Abundance of immune cell populations identified by deconvolution analysis of WTA using immune cell matrix pipeline at day 3 and 7 post-inoculation from (**e-f**) islets, (**g-h**) PLN,s and (**i-j**) spleen of the vehicle and TYK2i-treated *RIP-LCMV-GP* mice.


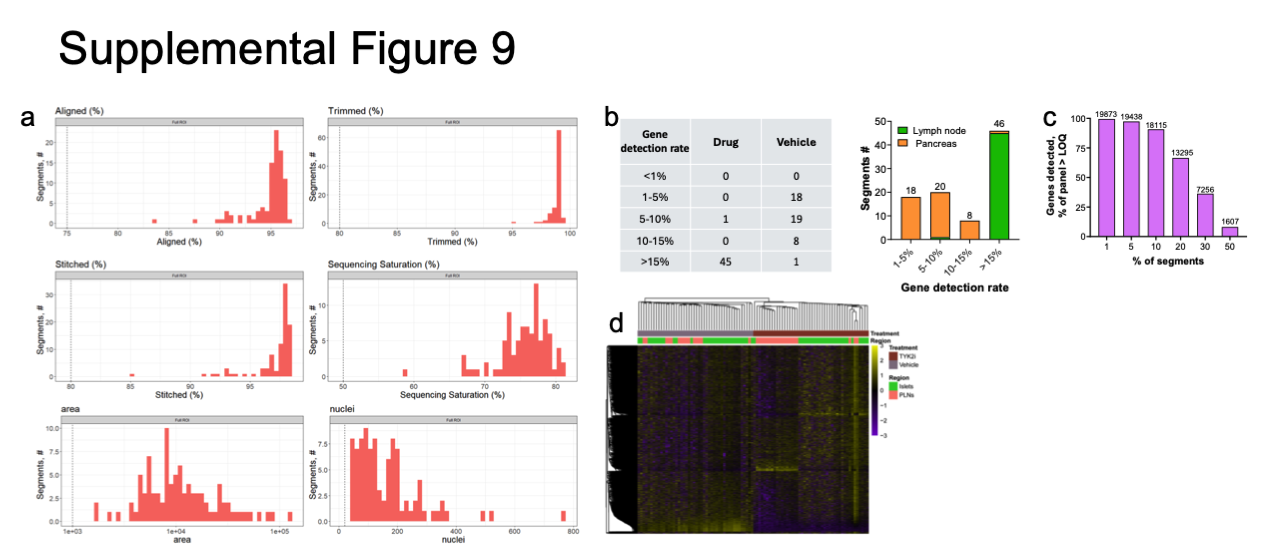


**Supplemental Figure 9. Quality control and normalization strategies used in spatial whole transcriptome analysis of NOD mouse.**

(**a**) Percentage of aligned sequencing read counts at every step of sequencing and sequencing saturation. (**b**) Gene detection rate across different cutoff ratios and (**c**) number of genes detected across different filtering criteria. (**d**) Heatmap showing gene clustering using genes with a high coefficient of variation from islets and PLN of vehicle- and TYK2-treated NOD mice.


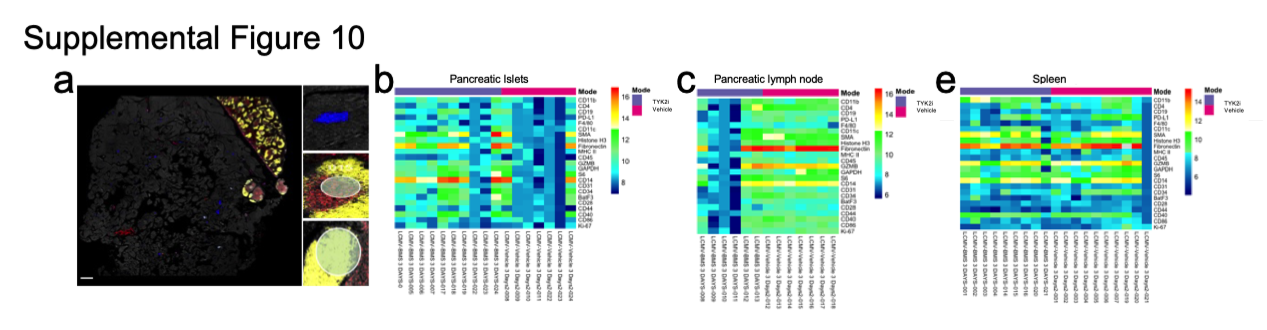


**Supplemental Figure 10. Spatial proteomics of islets, PLN, and spleen of *RIP-LCMV-GP* mice treated with vehicle or TYK2 inhibitor at day 3.**

(**a**) Representative images of islets, PLN, and spleen of vehicle- or TYK2i-treated *RIP-LCMV-GP* mice at day 3 post-inoculation labeled for CD3 (red), PTPRC (yellow), insulin (blue), and sytox83 (grey). (**b-d**) Heatmap showing the overall expression of immune cell typing and immune cell activation markers from selected regions of interest (ROIs) from (**b**) islets, (**c**) PLN, and (**d**) spleen; n=3 mice/group. Data were normalized to the geometric mean of the IgG negative control, and the significantly expressed proteins were presented as Log_2_ of signal-to-noise ratio (SNR).
