# Supplementary material for: Pharmacological inhibition of tyrosine protein-kinase 2 reduces islet inflammation and delays type 1 diabetes onset in mice
Source: eBioMedicine. 2025 May 6;117:105734. doi: 10.1016/j.ebiom.2025.105734 (PMC12173048; doi:10.1016/j.ebiom.2025.105734)
Supplement: Supplemental Western Blots [file mmc2.pptx]

## Slide 1
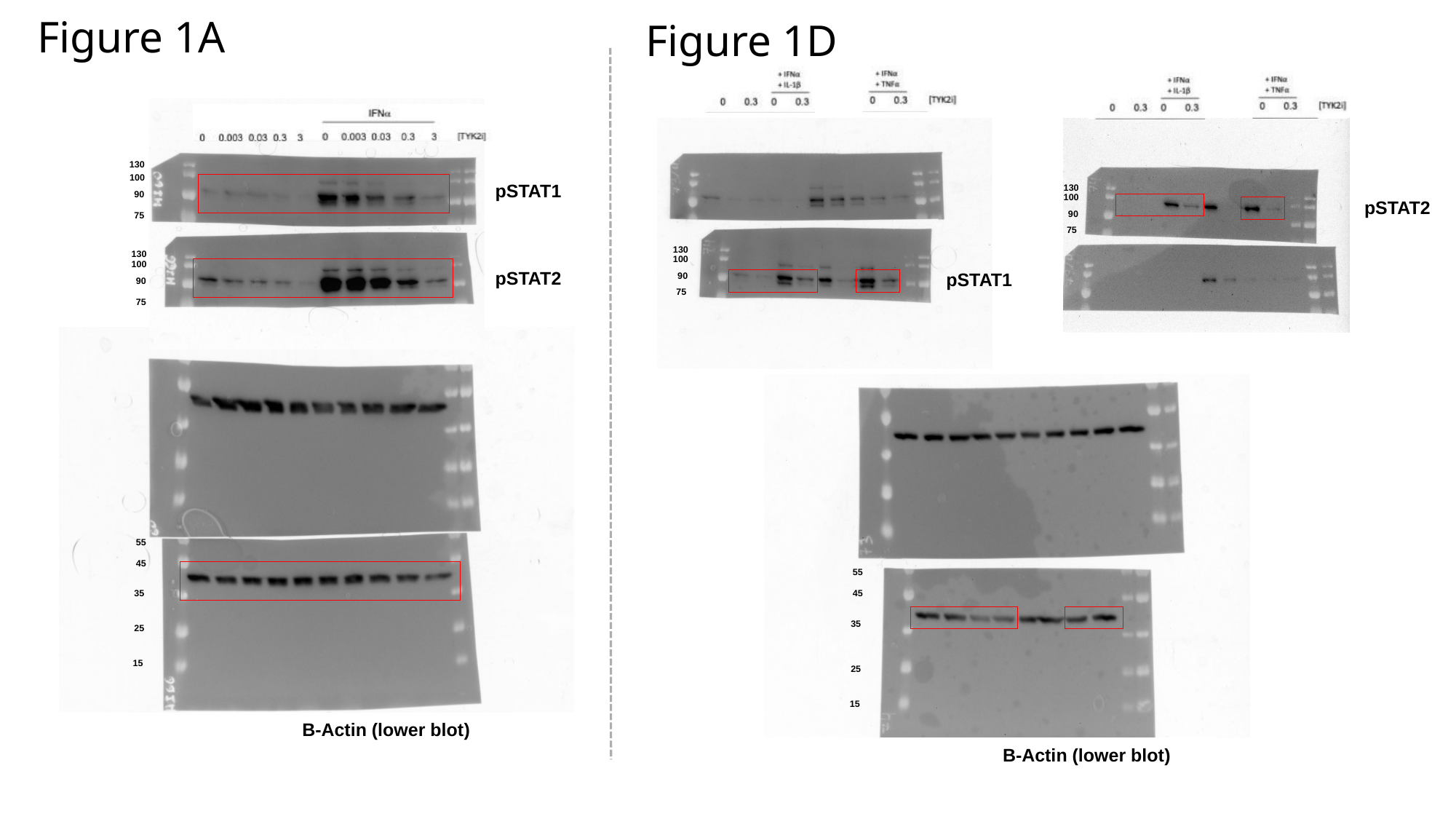

Figure 1A
Figure 1D
130
100
pSTAT1
130
90
100
pSTAT2
90
75
75
130
130
100
100
pSTAT2
pSTAT1
90
90
75
75
55
45
55
35
45
35
25
15
25
15
B-Actin (lower blot)
B-Actin (lower blot)

## Slide 2
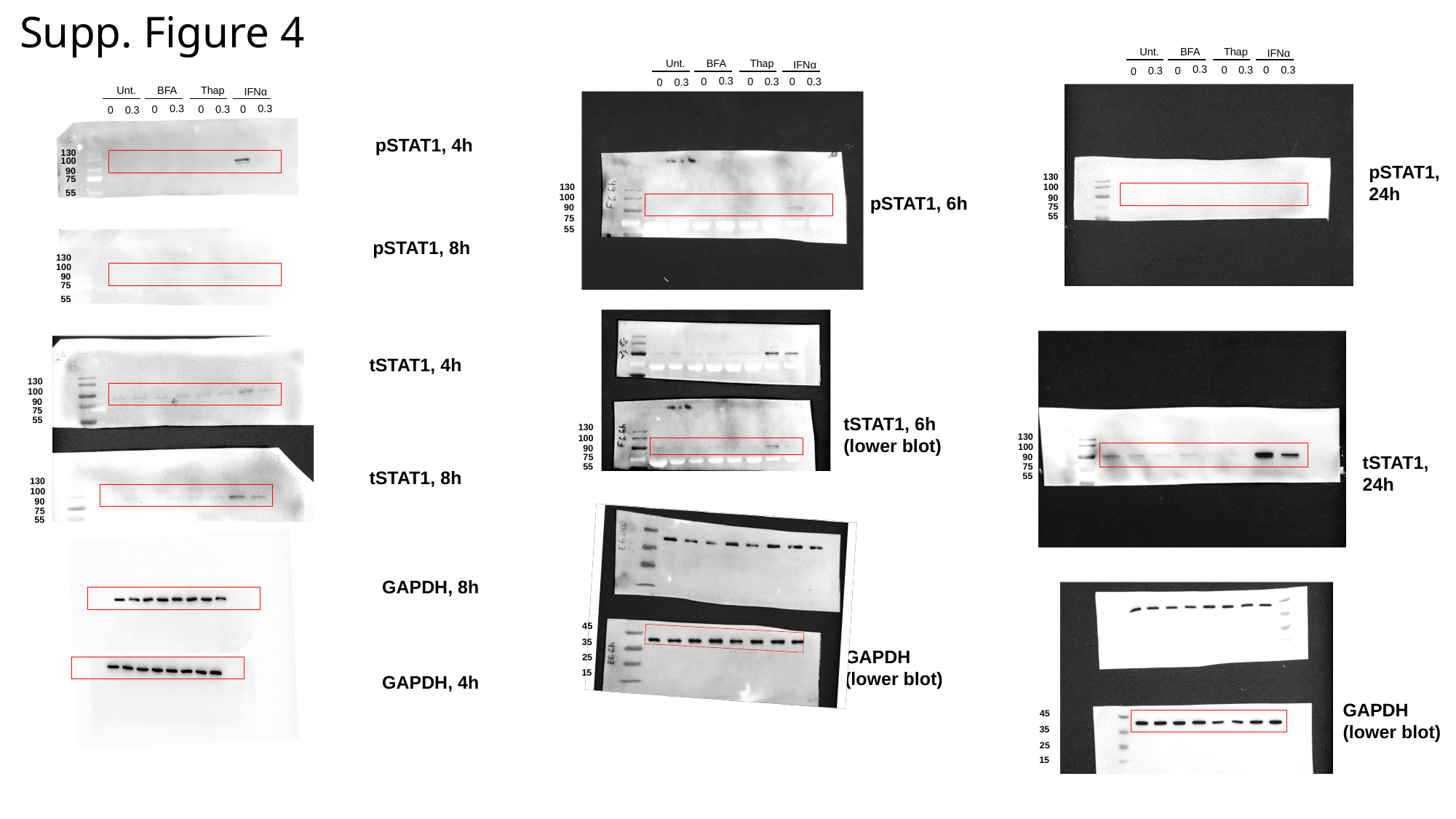

Supp. Figure 4
Unt.
BFA
Thap
IFNα
0.3
0.3
0
0.3
0
0
0.3
0
Unt.
BFA
Thap
IFNα
0.3
0.3
0
0.3
0
0
0.3
0
Unt.
BFA
Thap
IFNα
0.3
0.3
0
0.3
0
0
0.3
0
pSTAT1, 4h
130
100
pSTAT1,
24h
90
130
75
130
100
55
100
pSTAT1, 6h
90
75
90
55
75
55
pSTAT1, 8h
130
100
90
75
55
tSTAT1, 4h
130
100
90
75
tSTAT1, 6h
(lower blot)
55
130
130
100
100
90
tSTAT1,
24h
90
75
75
55
tSTAT1, 8h
55
130
100
90
75
55
GAPDH, 8h
45
35
GAPDH
(lower blot)
25
15
GAPDH, 4h
GAPDH
(lower blot)
45
35
25
15
