# Supplementary material for: Pharmacological inhibition of tyrosine protein-kinase 2 reduces islet inflammation and delays type 1 diabetes onset in mice
Source: eBioMedicine. 2025 May 6;117:105734. doi: 10.1016/j.ebiom.2025.105734 (PMC12173048; doi:10.1016/j.ebiom.2025.105734)
Supplement: Reagent validation [file mmc3.docx]

**Reagent validation**

☐ Please indicate how and whether cell lines have been validated.

☐ Please indicate how and whether antibodies have been validated.

☐ **Authors must clearly indicate whether and how antibody and cell lines have been validated, by providing a statement in the Methods section.**Please provide relevant documentation in the Supplemental Data (Reagent Validation file).

**Cell line validation**:

Recent STR profiling of cell lines is preferred, either by the lab/institute, or by commercial vendor or cell line repository, with relevant documentation. Please also indicate whether recent mycoplasma testing has been performed. Additional information and guidelines on cell line validation from the ICLAC can be found here:

[https://iclac.org/category/resources/](https://nam12.safelinks.protection.outlook.com/?url=https%3A%2F%2Fc05y1x9s.r.us-east-2.awstrack.me%2FL0%2Fhttps%3A%252F%252Ficlac.org%252Fcategory%252Fresources%252F%2F1%2F010f01928f71b800-31b7433c-c98e-4cd8-93ab-d4286ea504b6-000000%2FGAfisTFvT9yICnEd2HvtVyeodoI%3D180&data=05%7C02%7Candeemil%40iu.edu%7C9e6a51112f4946a1327a08dcede668d1%7C1113be34aed14d00ab4bcdd02510be91%7C0%7C0%7C638646821084468046%7CUnknown%7CTWFpbGZsb3d8eyJWIjoiMC4wLjAwMDAiLCJQIjoiV2luMzIiLCJBTiI6Ik1haWwiLCJXVCI6Mn0%3D%7C0%7C%7C%7C&sdata=24BijFlJo9UzZioo9W9mZ5waf5v6%2BpkIE74RKCFcjmg%3D&reserved=0)

[https://iclac.org/](https://nam12.safelinks.protection.outlook.com/?url=https%3A%2F%2Fc05y1x9s.r.us-east-2.awstrack.me%2FL0%2Fhttps%3A%252F%252Ficlac.org%252F%2F1%2F010f01928f71b800-31b7433c-c98e-4cd8-93ab-d4286ea504b6-000000%2F0xWuF978Xq2wojb8pSZX0QnQdx0%3D180&data=05%7C02%7Candeemil%40iu.edu%7C9e6a51112f4946a1327a08dcede668d1%7C1113be34aed14d00ab4bcdd02510be91%7C0%7C0%7C638646821084487484%7CUnknown%7CTWFpbGZsb3d8eyJWIjoiMC4wLjAwMDAiLCJQIjoiV2luMzIiLCJBTiI6Ik1haWwiLCJXVCI6Mn0%3D%7C0%7C%7C%7C&sdata=eKm4HB0YRsIoJRKW3WzQg%2B8q5xRU5Y5xe4I0%2BNr5SoQ%3D&reserved=0)

**Antibody validation**:

For commonly used, commercial antibodies--RRID tags and relevant references for application should be provided whenever possible. For non-commercial antibodies, including those generated in-house: please clearly indicate how the antibody was validated, including tests done for specificity and reactivity within the experimental system for which they are being used in the study.

 Examples of antibody validation as outlined by International Working Group for Antibody Validation can be found here:

 Uhlen, M., Bandrowski, A., Carr, S. et al. A proposal for validation of antibodies. Nat Methods 13, 823–827 (2016). [https://doi.org/10.1038/nmeth.3995](https://nam12.safelinks.protection.outlook.com/?url=https%3A%2F%2Fc05y1x9s.r.us-east-2.awstrack.me%2FL0%2Fhttps%3A%252F%252Fdoi.org%252F10.1038%252Fnmeth.3995%2F1%2F010f01928f71b800-31b7433c-c98e-4cd8-93ab-d4286ea504b6-000000%2FA6U4jzq2dTCZZgjNc9T1QAKo-6w%3D180&data=05%7C02%7Candeemil%40iu.edu%7C9e6a51112f4946a1327a08dcede668d1%7C1113be34aed14d00ab4bcdd02510be91%7C0%7C0%7C638646821084503017%7CUnknown%7CTWFpbGZsb3d8eyJWIjoiMC4wLjAwMDAiLCJQIjoiV2luMzIiLCJBTiI6Ik1haWwiLCJXVCI6Mn0%3D%7C0%7C%7C%7C&sdata=9G4t7ZX4faeVSjoFh%2F%2BMorNbv%2F59tE71jDoDP34U4ok%3D&reserved=0)

☐ Compound structures must be provided if previously unpublished. Please provide reference for published structures.

**Text added to manuscript:**

**Reagent Validation**

*Cell lines and iPSC cells*

EndoC-βH1 cells

Human insulin-secreting EndoC-βH1 cells (RRID:CVCL_L909) were kindly provided by Dr. R. Scharfmann, Institut Cochin, University of Paris, France, and are available commercially (<https://www.humancelldesign.com/endoc-bh1/>). According to the vendor, this high-quality human pancreatic β cell line displays a homogeneity that exceeds 99% and an insulin content of 0.5 to 1 µg/million cells. Functionality of EndoC-βH1 cells has been validated using glucose stimulated insulin secretion (GSIS) assays, and these production and assay processes are conducted with rigor and precision, in full respect of the industry’s highest quality standards for reliability and reproducibility. Cells are continuously monitored for doubling times and morphology at the Eizirik laboratory. In addition, they are tested every 9-12 months to ensure identity and verify they are free of contamination from other cell lines and microbes. The presence of mycoplasma infection was regularly controlled using the MycoAlert Mycoplasma Detection kit (Lonza). They were latest tested for mycoplasma on December 18, 2024. Cells are periodically refreshed from frozen stocks to maintain a low passage number.

iPSC Lines

Cell lines utilized for this project include the previously generated iPSC lines (generated by Eizirik lab) and newly de-differentiated iPSC generated from archived PBMC. For the generation of iPSC, episomal vectors are used to re-program fibroblasts or PBMCs. iPSCs are checked for the pluripotency markers OCT4, SSEA4, and TRA1-60, must have exogenous transgenes silenced (demonstrated by absent PCR amplification of the oriP/EBNA-1 backbone of the episomal vector), and must successfully differentiate into endoderm, mesoderm, and ectoderm germ layers in an embryoid body assay, and have a normal 46, XY or XX karyotype. Cultured lines are limited to 20 total passages, continuously monitored for doubling times and morphology, and tested periodically for contamination using standard detection kits. They were latest tested for mycoplasma on December 18, 2024. Cell lines are authenticated periodically by sequencing.

*iPSC-derived β cells*: iPSC lines used for generating β cells consistently progress through 7-stages of differentiation into pancreatic β cells, must follow a pattern consistent with pancreatic, endocrine, and β cell development, reaching mRNA levels of insulin and Pdx1 that are overall comparable to primary human islets, usually resulting in >50% β cells and few α- or insulin- and glucagon-double positive cells. Islet identity and quality are assessed in a stepwise fashion by performing PDX1 and NKX6.1 staining to confirm pancreatic progenitor identity, performing imaging to assess the distinctness of insulin and glucagon staining in mature islet clusters, and by verifying glucose stimulated insulin secretion.

*Antibodies:*

All antibodies used in this study were procured from commercial vendors. A detailed list of the antibodies, along with their associated RRIDs, is provided in the accompanying Excel file.
